# Bromodomain and extraterminal protein inhibitor JQ1 induces maturation arrest and disrupts the cytoplasmic organization in mouse oocytes under in vitro conditions

**DOI:** 10.1038/s41598-025-96687-z

**Published:** 2025-04-18

**Authors:** Keerthana Karunakar Poojary, Jyolsna Ponnaratta Kunhiraman, Vanishree Vasave Madhvacharya, Sandhya Kumari, Navami Krishna, Suresh P S, Rajanikant G K, Srinivas Mutalik, Nadeem Khan Ghani, Shama Prasada Kabekkodu, Thottethodi Subrahmanya Keshava Prasad, Satish Kumar Adiga, Guruprasad Kalthur

**Affiliations:** 1 Centre of Excellence in Clinical Embryology, Department of Reproductive Science, Kasturba Medical College, Manipal, Manipal Academy of Higher Education, Manipal, 576104 India; 2 Division of Reproductive Biology, Department of Reproductive Science, Kasturba Medical College, Manipal, Manipal Academy of Higher Education, Manipal, 576104 India; 3 Department of Bioscience and Engineering, National Institute of Technology, Calicut, 673601 Kerala India; 4 Department of Pharmaceutics, Manipal College of Pharmaceutical Sciences, Manipal Academy of Higher Education, Manipal, 576104 India; 5 Manipal School of Life Sciences, Manipal Academy of Higher Education, Manipal, 576104 India; 6 Center for Systems Biology and Molecular Medicine [An ICMR-Collaborating Centre of Excellence (ICMR-CCoE 2024)], Yenepoya Research Centre, Yenepoya (Deemed to Be University), Mangalore, 575018 India

**Keywords:** In vitro maturation, Mitochondrial distribution, Spindle abnormalities, Symmetric cytokinesis, Reactive oxygen species, BRD4, Cell biology, Developmental biology, Endocrinology

## Abstract

JQ1, a small cell-permeable molecule is known for its potent inhibitory action on bromodomain and extraterminal (BET) proteins. Although earlier studies have shown its inhibitory effect on male gametogenesis, limited information is available about its influence on oocyte development. Since BET genes are known to exhibit regulatory functions on oocyte development and maturation, the present study aimed to investigate the effect of JQ1 on oocyte developmental competence under in vitro conditions*.* Germinal vesicle (GV) stage oocytes were collected from adult Swiss albino mice and subjected to in vitro maturation (IVM) in the presence of various concentrations of JQ1 (25, 50, and 100 μM). The metaphase II (MII) stage oocytes were assessed for cytoplasmic organization and functional competence at 24 h after IVM. A significant decrease in nuclear maturation (at 50 and 100 μM), symmetric cytokinesis, altered distribution of mitochondria and cortical granules, poorly organized actin and meiotic spindle, misaligned chromosomes, and elevated endoplasmic reticulum (ER) stress and oxidative stress was observed in JQ1-exposed oocytes. Presence of N-acetyl cysteine (NAC), in IVM medium resulted in significant reduction in JQ1-induced oxidative stress and symmetric cytokinesis. Administration of JQ1 (50 mg/kg, intra peritoneal) to adult Swiss albino mice primed with pregnant mare serum gonadotrophin (PMSG) and human chorionic gonadotrophin (hCG) did not affect the ovulation. However, a high degree of oocyte degeneration, elevated intracellular reactive oxygen species (ROS), and GRP78 expression was observed in JQ1-administered mice. In conclusion, our study reveals that BET inhibitor JQ1 has detrimental effects on oocyte function and development.

## Introduction

The bromodomain and extra-terminal (BET) family of proteins are known as epigenetic readers that specifically bind to acetyl-lysine recognition motifs on histones, regulating chromatin remodeling^1^. Based on their structural similarities, bromodomains are classified into BRD2, BRD3, BRD4, and the testes-specific BRDT^2,3^. These proteins have been implicated in the pathogenesis including cancer^4^, cardiovascular diseases^5^, musculoskeletal disorders, inflammation, and neurological diseases^1,6^. Further, studies have reported that BET family proteins are expressed in somatic and germ cells and play significant roles in oocyte development^7^, spermatogenesis^8^ and fetal growth^9^.

BET proteins have gained significant attention in recent years as potential targets for identifying novel inhibitors to treat a wide range of pathological conditions. JQ1, [(4-(4-chlorophenyl)-2,3,9-trimethyl-1,1-dimethylethylester-6H-thieno(3,2-f) (1,2,4)-triazolo(4,3-a)-(1,4)diazepine-6S-acetic acid)], is a potent BET inhibitor, that can easily permeate cell membranes. It competitively binds to the acetyl-lysine recognition motifs of the bromodomain proteins, inhibiting cell proliferation and differentiation^10^. They also demonstrated that JQ1 can bind to all members of the BET family proteins, with a high affinity for BRD4. JQ1 has been used to treat pathological conditions such as neurodegenerative diseases^11^ and cancer^12^.

An earlier study by Matzuk et al.^13^ demonstrated that JQ1 can act as a reversible male contraceptive molecule. Further, a recent study by Wang et al.^14^ revealed that the inhibitory effect of JQ1 on spermatogenesis is mediated through altering the chromatin architecture of the germ cells. Experiments on embryonic stem cells have shown that JQ1 exposure significantly decreases cell proliferation and the expression of *Nanog, Sox2,* and *Oct4*^15^. Considering the role of BET genes in oocyte development, and the ability of JQ1 to inhibit pluripotency genes involved in oocyte development, JQ1 might have adverse effects on the oocyte function. However, there are no studies in the literature to address these aspects. Therefore, the present study was aimed at assessing the impact of the broad-spectrum BET inhibitor JQ1 on oocyte functional competence under in vitro conditions.

## Materials and methods

### Animals

The experiments were conducted using inbred adult female Swiss albino mice (6–8 weeks) housed in the Central Animal Research Facility at Kasturba Medical College, Manipal Academy of Higher Education, Manipal. The mice were kept under standard conditions of humidity (45–55%), temperature (25 ± 2 °C), and a 12:12 h light–dark cycle, with food and water ad libitum. The study was approved by the Institutional Animal Ethics Committee of Kasturba Medical College, Manipal (IAEC/KMC/61/2020). Institutional guidelines and the guidelines of the Committee for the Purpose of Control and Supervision of Experiments on Animals (CPCSEA) were strictly followed for animal handling, and the reporting of animal experiments follows ARRIVE (Animal Research: Reporting of In vivo Experiments) guidelines.

### In vitro maturation (IVM)

The IVM media was freshly prepared using Dulbecco’s Modified Eagle Medium (DMEM, Cat. No. D5648, Sigma Aldrich, USA) supplemented with 1% insulin transferrin selenium (ITS; Cat. No. 51500056, Gibco™, USA), 1% non-essential amino acids (Cat. No. 11140050, Gibco™, ThermoFisher Scientific, USA), 0.05% sodium pyruvate (Cat. No. P3662, Sigma Aldrich, USA), and 0.3% bovine serum albumin (BSA; Cat. No. A3311, Sigma Aldrich, USA). JQ1 stock solution (1 M) was prepared in DMSO (dimethyl sulfoxide; Cat. No. 5879, Sigma Aldrich, USA) and stored at −20 °C. The working solutions of 25, 50 and 100 µM concentrations were freshly prepared before each experiment by diluting the stock solution in IVM medium. For the vehicle control group 0.1% of DMSO was used for the experiment.

### Germinal vesicle (GV) stage oocyte collection

**Fig. 1:**
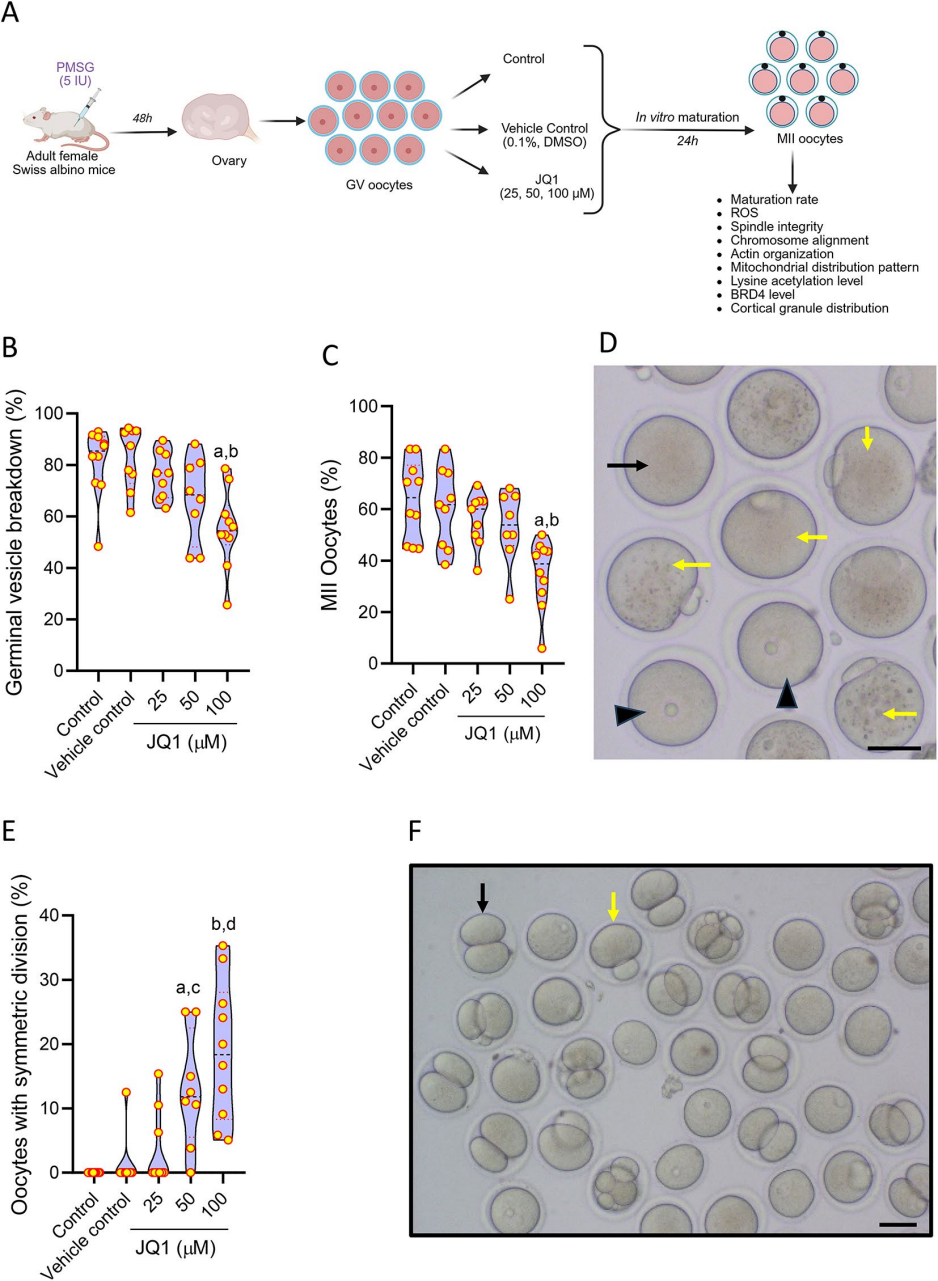
(**A**) Schematic representation of the study outline to assess the effect of JQ1 on the nuclear maturation and cytoplasmic function of oocytes under in vitro conditions (Created with BioRender.com). (**B**) Effect of different concentrations of JQ1 on in vitro maturation of germinal vesicle (GV) stage oocytes collected from Swiss albino mice at 24 h after culture. GVBD rate at 24 h after in vitro maturation in GV oocytes cultured in presence of various concentrations of JQ1. ^a^p < 0.001 compared to control; ^b^p < 0.001 compared to vehicle control. (**C**) Maturation (MII) rate of GV stage oocytes at 24 h after in vitro culture. ^a^p < 0.001 compared to control; ^b^p < 0.01 compared to vehicle control. (**D**) Representative images of oocytes at different stages of nuclear maturation. The black arrowhead indicates GV stage oocyte; black arrow indicates MI oocyte; yellow arrow indicates MII stage oocytes (magnification 100x). The scale bar represents 100 µm. (**E**) Percentage of oocytes exhibiting symmetric cytokinesis during in vitro maturation at 24 h after culture. ^a^p < 0.01, ^b^p < 0.0001 compared to control; ^c^p < 0.05, ^d^p < 0.0001 compared to vehicle control. (**F**) Representative images of oocytes subjected to IVM in the presence of JQ1 at 24 h. Yellow arrow indicates oocytes with asymmetric cytokinesis; black arrow indicates oocyte with symmetric cytokinesis (magnification 100x). The scale bar represents 50 µm. Number of oocytes in Control = 235; Vehicle control = 192; JQ1 25 µM = 244; JQ1 50 µM = 204; JQ1 100 µM = 334.

Healthy adult female Swiss albino mice (6–8 weeks old) were primed with 5 IU pregnant mare serum gonadotropin (PMSG, Cat. No. HOR-272, ProSpec-Tany TechnoGene Ltd., Israel). At 48 h after PMSG administration, the mice were humanely sacrificed by cervical dislocation, and the ovaries were collected in DMEM. GV stage oocytes, free of granulosa cells, were gently teased from the ovaries using a blunt needle in DMEM containing 0.3% BSA. The oocytes were then randomly divided into control (IVM media), vehicle control (0.1% DMSO in IVM media), and three concentrations of JQ1 (25, 50, and 100 µΜ, respectively, in IVM media). After 24 h of incubation at 37 °C and 5% CO_2_, oocytes were assessed for nuclear maturation using an inverted microscope (Olympus IX73, Japan) with a stage warmer maintained at 37 °C (Fig. 1A). The data for Germinal vesicle breakdown (GVBD) and maturation rate (MII oocytes) were represented in percentages^16^.

### Spindle organization and chromosome alignment

Spindle organization in IVM-derived MII oocytes was assessed following the method described by Hegde et al.^17^. Briefly, MII oocytes were transferred to extraction buffer and incubated at 37 °C for 60 min. Subsequently, they were fixed with chilled methanol (−20 °C) for 12 min. Afterward, the oocytes were placed in blocking solution (PBS containing 5% BSA) at 37 °C for 1 h, followed by overnight incubation with primary monoclonal anti α-tubulin antibody (Cat. No. T9026, Sigma Aldrich, USA) at 4 °C, and 1 h incubation with secondary antibody (goat anti-mouse IgG FITC, Cat. No. NB7538, Novus biologicals, USA) at 37 °C. The oocytes were washed in PBS and counterstained with 4′,6′-diamidino-2-phenylindole (DAPI; Cat. No. D9542, Sigma-Aldrich, USA), and examined under a fluorescence microscope (Axio Imager A1, Zeiss, Germany) and images were captured using Leica Application Suite X software (Leica microsystems, Germany). Oocytes exhibiting a bipolar spindle with centrally aligned chromosomes were considered to have a normal spindle organization, while those showing a distorted, broad, narrow, or no spindle were classified as having abnormal spindle patterns. MII oocytes with misaligned chromosomes at the equatorial plate were scored and the data was expressed in percentage^18^.

### Intracellular reactive oxygen species (ROS) level

Intracellular ROS level was assessed in MII oocytes derived from IVM. Briefly, the oocytes were incubated with 10 μM DCFH-DA (dichlorodihydro fluorescein diacetate, Cat. No. D6883, Sigma-Aldrich, USA) for 30 min at 37 °C and 5% CO_2_, followed by 3–4 washes in DMEM medium. Subsequently, the washed oocytes were immediately transferred to a clean glass slide and observed under a fluorescence microscope using a 405–435 nm filter. The ROS level was estimated based on the green fluorescence intensity using the Q-capture software (Q-Imaging, Surrey, Canada), and the percentage of oocytes exhibiting high, moderate, and low intensity was calculated^17^.

### Mitochondrial distribution pattern

The mitochondrial distribution pattern in MII oocytes was assessed using Rhodamine 123 dye (Cat. No. R8004, Sigma Aldrich, USA), following the method described by Hegde et al. ^17^. Briefly, oocytes were incubated in DMEM culture media containing 10 µg/mL of Rhodamine 123 for 20 min at 37 ℃ and 5% CO_2_, followed by thorough washing in DMEM media. Subsequently, the oocytes were transferred onto a clean glass slide and observed under a fluorescence microscope (Axio Imager A1, Zeiss, Germany). Based on the distribution pattern of mitochondria, the percentage of oocytes with uniformly distributed or aggregated mitochondrial distribution patterns were calculated and expressed in percentage.

### Actin distribution pattern

Actin filament distribution assessment and staining were performed following the method described by Hegde et al. ^17^. Briefly, MII oocytes were washed in PBS and fixed overnight in 4% paraformaldehyde (PFA) at 4 ℃. Subsequently, the oocytes were thoroughly washed in PBS containing 1% BSA and incubated for 30 min at room temperature with a 0.5 x concentration of Phalloidin-iFluor 488 (Cat. No. ab176753, Abcam, USA). After three washes in PBS containing 1% BSA, the oocytes were mounted on clean glass slides, counterstained with DAPI, and viewed under a fluorescence microscope (Axio Imager A1, Zeiss, Germany). Based on the actin organization pattern, percentage of oocytes with uniform or punctate actin organization was calculated.

### Cortical granule distribution

Cortical granule distribution in oocytes was assessed using Lens Culinaris Agglutinin (LCA) staining, as described in our earlier study^19^. Briefly, the oocytes were fixed in 4% PFA in PBS overnight at 4 °C. Subsequently, the oocytes were incubated in extraction buffer overnight at 4 °C, followed by incubation in blocking buffer (1% BSA, 0.01 M glycine, and 0.05% Triton X-100) at 37 °C for 1 h. The oocytes were then stained with 10 µg/mL of Rhodamine-LCA (Cat. No. RL-1042, Vector Laboratory, Inc., CA, USA) for 30 min at room temperature and thoroughly washed with PBS. After counterstaining with DAPI, the oocytes were mounted using Fluoroshield mounting medium (Cat. No. F6182, Sigma-Aldrich, USA). Oocytes were observed under confocal microscope (Leica SP8-DMi8 microscope, Germany), and the images were captured at 63 x oil immersion objective (630 x magnification) using Leica Application Suite X software. The percentage of oocytes with normal (peripheral and homogenous distribution) and abnormal (non-homogenous distribution) was calculated.

### Expression of glucose regulated protein 78 (GRP78), BRD4, global protein lysine acetylation and phosphorylated mTOR (p-mTOR) expression in MII oocytes

The IVM-derived MII oocytes were assessed for GRP78 and BRD4 expression, and lysine acetylation and p-mTOR level in MII oocytes by immunofluorescence. Briefly, oocytes were fixed in 4% PFA in PBS at room temperature for 30 min, followed by permeabilization in 0.2% Triton X-100 in PBS for 10 min. Subsequently, the oocytes were incubated in a blocking solution (5% BSA in PBS) for 1 h, followed by overnight incubation at 4 °C with appropriate primary antibodies (Acetylated lysine, 1:1000, Cat. No. 9441, Cell Signaling Technology, USA; BRD4, 1:500, Cat. No. PA5-100998, Invitrogen, ThermoFisher Scientific, USA; p-mTOR, 1: 1000, Cat. No. 29835, Cell Signaling Technology, USA, Anti-GRP78, 1: 1000; Cat. No. SAB4501452, Sigma Aldrich, USA) and corresponding secondary antibody (Goat Anti-Rabbit IgG Alexa Fluor 488, Cat. No. ab150077, Abcam, UK) for 1 h at 37 °C. The oocytes were counterstained with DAPI. Oocytes were observed under fluorescence microscope (Axio Imager A1, Zeiss, Germany), images were captured using Leica Application Suite X software (Leica microsystems, Germany). The GRP78, BRD4, lysine acetylation and p-mTOR levels were assessed based on the fluorescence intensity (Lum) using the Q-capture software (Q-Imaging, Surrey, Canada).

### Effect of N-acetyl cysteine (NAC) on intracellular ROS

NAC was employed to investigate whether the adverse effects on oocyte quality observed in the JQ1-exposed group were mediated through the generation of ROS. GV oocytes collected were randomly divided into four groups: control (IVM media), NAC (0.6 mM of NAC in IVM), JQ1 (100 µM in IVM), and NAC + JQ1 (0.6 mM NAC in IVM medium with 100 µM JQ1). ROS assessment was done in GV stage oocytes at 1 h after exposure and in MII oocytes at 24 h after exposure (Fig. 6C). The concentration of NAC used in this study was based on previous report^20^.

### Effect of JQ1 on induced ovulation in mice

To investigate whether JQ1 interferes with ovulation, adult female Swiss albino mice were subjected to superovulation using PMSG (5 IU, Cat. No. HOR-272, ProSpecTany TechnoGene Ltd., Israel), and 48 h later with human chorionic gonadotrophin (hCG, 10 IU, Lupin, India). After 30 min of hCG trigger, the mice were injected with 50 mg/kg of JQ1 by intraperitoneal route. At 12 h after hCG administration, the oocyte cumulus complexes (OCCs) were collected from oviduct in DMEM media. The oocytes were stripped off from the cumulus cells by incubating them for 30 s in hyaluronidase (Cat. No. H4272, Sigma Aldrich, USA, 0.75 mg/mL) at 37 ℃, followed by mechanical pipetting. After denudation, the number of MII oocytes, degree of fragmentation, intracellular ROS level, and GRP78 expression level were assessed (Fig. 8A).

### DNA damage assessment in cumulus cells by γH2AX assay

Cumulus cells were collected after the denudation of OCCs. The cells were washed with PBS at 500 g for 10 min, followed by overnight fixation with 4% PFA at 4 °C. The cells were washed with PBS by centrifugation at 500 g for 10 min. The supernatant was discarded, and the pellet was resuspended in permeabilization solution (PBS containing 0.1% triton X-100) and incubated at room temperature for 10 min. The cells were washed with PBS by centrifugation at 500 g for 10 min and the pellet was incubated with a blocking solution (PBS with 5% BSA) for 1 h. Followed by incubation of cells overnight at 4 °C with γH2AX primary antibody (1:500, Cat. No. 2577S, Cell Signalling Technology). The sample was washed by centrifugation at 500 g for 10 min to remove excess antibodies and incubated with secondary antibody (1: 1000, Goat Anti-Rabbit IgG Alexa flour 488, Cat. No. ab150077, Abcam, UK) for 1 h at 37 °C. The cells were then washed with PBS by centrifugation at 500 x g for 10 min. The cells were counterstained with DAPI and visualized under fluorescence microscope under 100 x magnification. Cells expressing γH2AX were scored in at least 500 cells and expressed as percentage of γH2AX-positive cells.

### Estimation of estradiol in serum

Estradiol in blood serum was estimated using the ELISA kit (DEMEDITEC, Cat. No. DEH3355, Germany). Blood collected from mice by cardiac puncture was transferred into a tube and allowed to stand at room temperature for 2 h to facilitate serum separation. The serum was then collected by centrifugation at 556 g, and the top layer was collected and stored at −80 °C until further processing. 25 µL of either standard or serum sample was added to the wells and incubated for 60 min at room temperature on a plate shaker maintained at > 600 rpm, followed by incubation for 60 min at room temperature with 100 μL of enzyme conjugate. The plates were then washed four times and incubated with 200 μL of substrate solution for 30 min in the dark. The reaction was then stopped by the addition of 50 μL of the stop solution. The optical density (OD) of the samples were measured at 450 nm immediately. The E2 level was determined by plotting the values on the standard curve generated from the reference standard provided in the ELISA kit.

### Gene enrichment and interaction network analysis of JQ1 target genes

In this study, JQ1 target genes were identified through molecular docking, with detailed procedures and results provided in the supplementary file. Subsequently, network enrichment analysis was conducted on JQ1 target genes with high binding affinity scores to elucidate the biological implications of gene expression changes induced by JQ1 treatment. To explore protein interactions with the target genes, we utilized STRING with specific parameters, such as a full STRING network and a high-confidence interaction score of 0.700. The relationships between JQ1-targeted proteins and the top 100 interacting genes were analyzed and visualized using Cytoscape^21^.

Concurrently, the ClueGO plugin from Cytoscape 3.7.1 was employed to visually analyze KEGG and GO functional enrichment^22,23^. The KEGG and GO pathway analyses were filtered for kappa > 0.74 and p < 0.01. ClueGO categorized non-redundant Gene Ontology (GO) terms and depicted functionally related genes in a clustered network^24^. In contrast, CluePedia expanded ClueGO’s functionality by integrating additional biological data to generate screened results and identify novel markers potentially linked to different pathways^25^.

### Statistical analysis

All the experiments were conducted at least twice. Each replicate was performed as an independent experiment. The results were represented as mean ± SEM. The graphs and statistical analysis were conducted using GraphPad Prism 10.4.1 (GraphPad Inc., USA). The data were analyzed by one way ANOVA, unpaired t-test, and Fisher’s exact test. The data with p < 0.05 was considered statistically significant.

## Results

### JQ1 induces meiotic arrest in oocytes during in vitro maturation

Oocytes were observed for nuclear maturation at 24 h after IVM. JQ1 did not have any significant effect on oocyte maturation at 25 and 50 µM concentrations. However, at 100 µM concentration, a significant reduction in GVBD (p < 0.001, Fig. 1B) and maturation (MII stage oocytes) rate (p < 0.001, Fig. 1C,D) was observed compared to control. Further, a dose-dependent increase in the percentage of MII oocytes with 2 cell stage embryo-like appearance (oocytes which underwent symmetric cytokinesis) were observed in JQ1 exposed oocytes, especially at 50 µM (p < 0.01) and 100 µM JQ1 (p < 0.0001, Fig. 1E,F) compared to control.

### JQ1 alters the mitochondrial distribution pattern and F-actin organization in MII oocytes

**Fig. 2:**
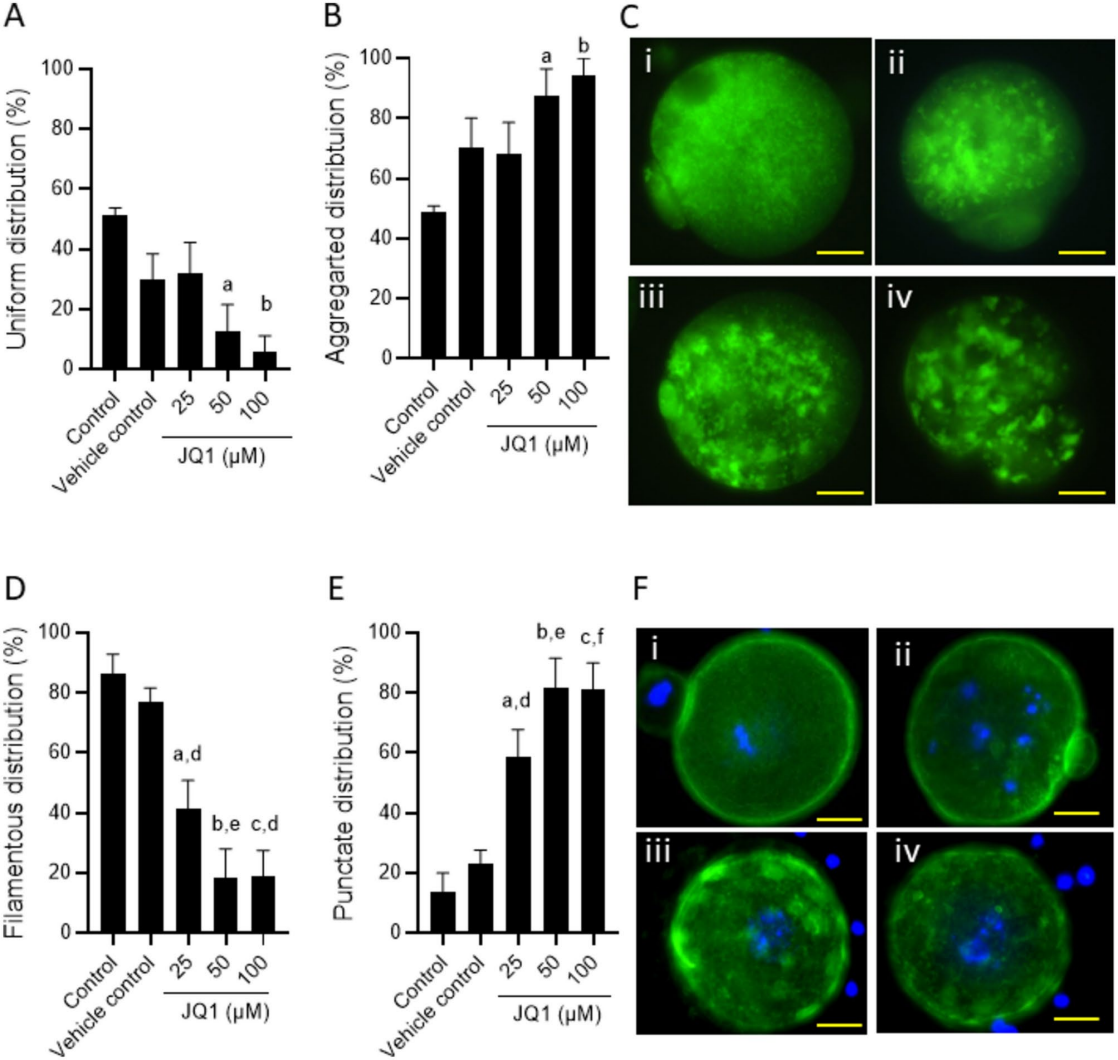
Effect of JQ1 on the mitochondrial distribution pattern assessed in MII stage oocytes at 24 h after IVM by Rhodamine 123 staining. Percentage of MII oocytes with (**A**) uniform and (**B**) aggregated mitochondrial distribution. ^a^p < 0.05, ^b^p < 0.01 compared to control. (**C**) Representative images showing MII oocytes with different distribution patterns of mitochondria; (i) uniform distribution pattern, (ii-iv) aggregated distribution pattern (magnification 400x). The scale bar represents 20 µm. Number of oocytes in Control = 58; Vehicle control = 44; JQ1 25 µM = 82; JQ1 50 µM = 55; JQ1 100 µM = 17. Effect of JQ1 exposure on actin organization pattern in MII oocytes assessed using Phalloidin-iFluor 488 staining method; Percentage of MII oocytes with (**D**) filamentous and (**E**) punctate organization. ^a^p < 0.05, ^b^p < 0.001, ^c^p < 0.0001 compared to control; ^d^p < 0.05, ^e^p < 0.01, ^f^p < 0.001 compared to vehicle control. (**F**) Representative images showing different actin filament organization in MII oocytes; (i) filamentous organization, (ii-iv) punctate organization (magnification 400x). The scale bar represents 20 µm. Number of oocytes, Control = 40; Vehicle control = 39; JQ1 25 µM = 32; JQ1 50 µM = 22; JQ1 100 µM = 27.

In the control group, 50% of MII oocytes had uniformly distributed mitochondria, (Fig. 2A). JQ1 altered the mitochondrial distribution pattern in oocytes as evidenced from an increase in the percentage of oocytes with aggregated (Fig. 2B,C) mitochondrial organization (25 µM: p < 0.05; 50 µM: p < 0.0001 and 100 µM: p < 0.001). Further, majority of MII oocytes in the control group (87%) and the vehicle control group (77%) exhibited filamentous organization of actin (Fig. 2D,F), while the rest displayed punctate organization (Fig. 2E,F). However, JQ1 exposure led to a significant increase (p < 0.0001) in the percentage of oocytes with punctate organization. At 25 µM concentration, the JQ1-exposed group had 58.49% of oocytes with punctate organization, which significantly (p < 0.001 compared to control) increased to 81.74% and 81.25% at 50 and 100 µM concentrations, respectively.

### JQ1 affects cortical granule distribution in MII oocytes

**Fig. 3:**
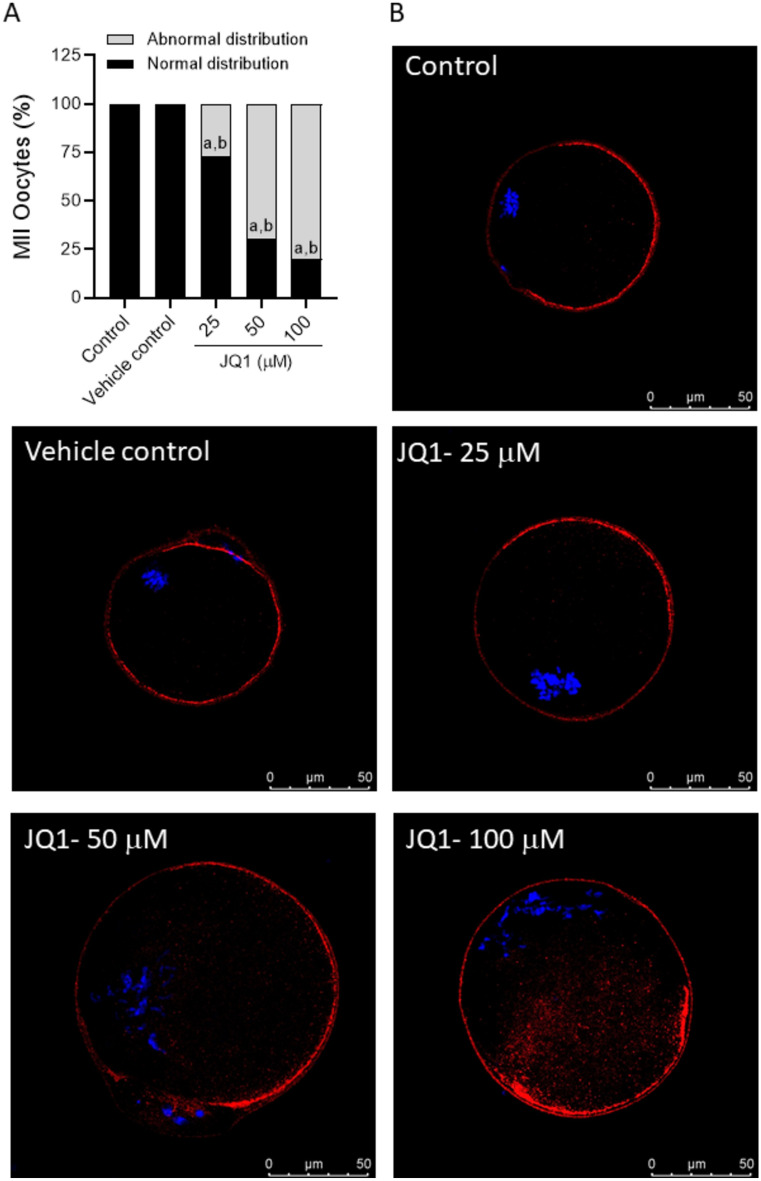
(**A**) Effect of JQ1 on the cortical granule distribution pattern in MII oocytes. ^a^p < 0.0001 compared to control; ^b^p < 0.0001 compared to vehicle control. (**B**) Representative (confocal) images of MII oocytes exposed to various concentrations of JQ1 during in vitro maturation stained with Rhodamine-Lens Culinaris Agglutinin (LCA) and counterstained with DAPI (magnification 630x). The scale bar represents 50 µm. Number of oocytes, Control = 15; Vehicle control = 12; JQ1 25 µM = 15; JQ1 50 µM = 13; JQ1 100 µM = 15.

In both the control and vehicle control groups, all the oocytes exhibited normal cortical granule distribution pattern (Fig. 3A,B). However, JQ1 exposure led to a significant increase (p < 0.0001) in the percentage of oocytes with abnormal cortical granule distribution, with the incidence increasing in a dose-dependent manner (26.67, 69.23, and 80% at 25, 50, and 100 µM concentrations of JQ1, respectively).

### JQ1 disrupts meiotic spindle assembly and chromosome alignment in MII oocytes

**Fig. 4:**
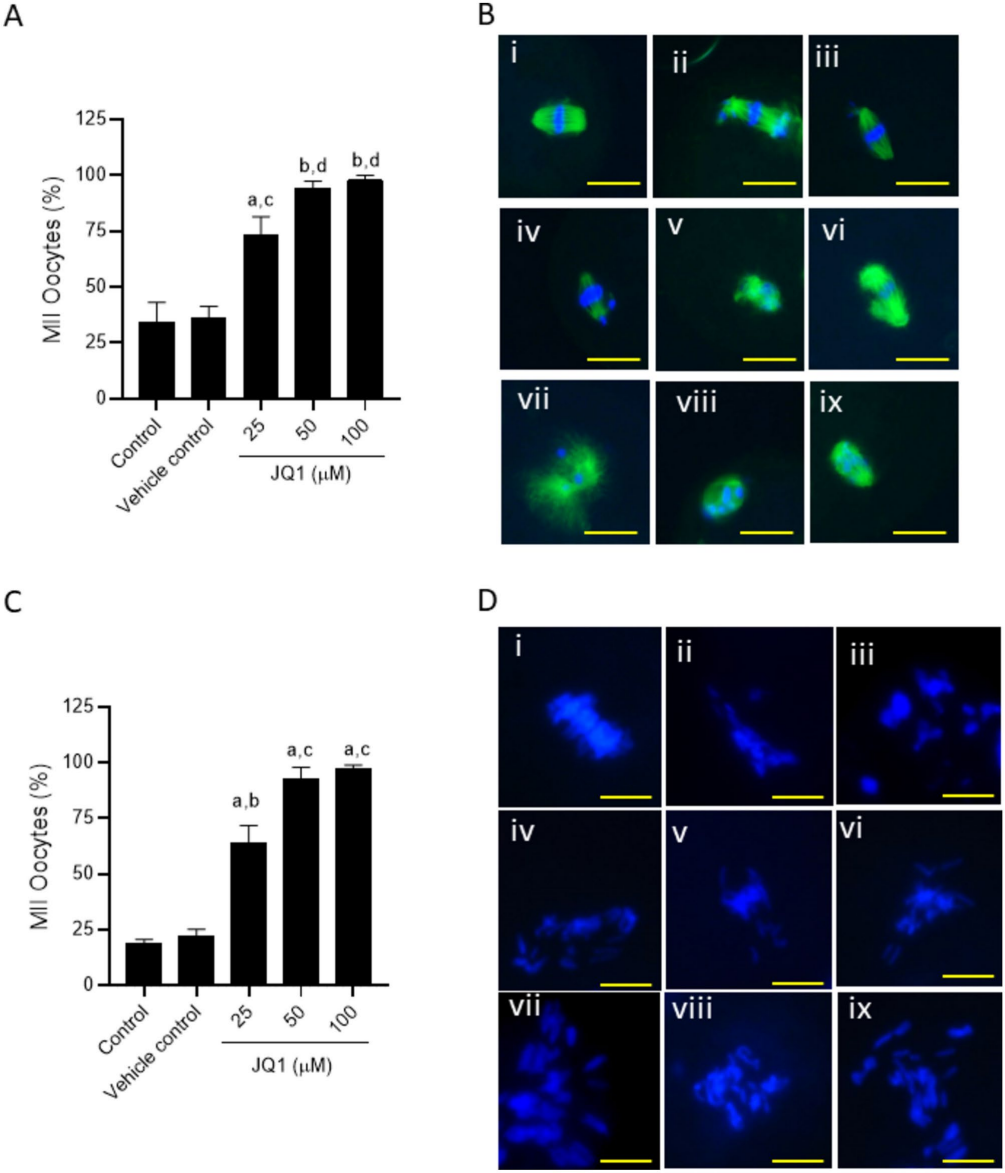
(**A**) Effect of JQ1 on the organization of meiotic spindle in MII oocytes. ^a^p < 0.01, ^b^p < 0.001 compared to control; ^c^p < 0.01, ^d^p < 0.001 compared to vehicle control. (**B**) Representative images of oocytes with (i) normal and (ii- ix) abnormal spindle organization were assessed by staining the spindle with anti-α tubulin antibody and counterstaining with DAPI (magnification 400x). The scale bar represents 20 µm. Number of oocytes in Control = 23; Vehicle control = 29; JQ1 25 µM = 31; JQ1 50 µM = 27; JQ1 100 µM = 38. (**C**) Effect of JQ1 on the chromosome alignment in MII stage oocytes. ^a^p < 0.0001 compared to control; ^b^p < 0.001, ^c^p < 0.0001 compared to vehicle control. (**D**) Representative images of chromosome alignment in MII stage oocytes; (i) normal chromosome alignment and (ii-ix) oocytes with misaligned chromosomes. The scale bar represents 10 µm. Number of oocytes in Control = 112; Vehicle control = 146; JQ1 25 µM = 159; JQ1 50 µM = 67; JQ1 100 µM = 101.

JQ1 significantly disrupted the spindle organization in MII oocytes at all the concentrations studied [80.64% at 25 µM (p < 0.01 compared to control and vehicle control)], 92.59% at 50 µM (p < 0.001 compared to control and vehicle control), and 100% at 100 µM (p < 0.001 compared to control and vehicle control] (Fig. 4A,B). Furthermore, oocytes exposed to JQ1 displayed a significantly higher percentage (p < 0.001 for 25 µM, p < 0.0001 for 50 and 100 µM) of MII oocytes with misaligned chromosomes at all concentrations of JQ1 compared to the MII oocytes from the control and vehicle control groups (Fig. 4C,D).

### Expression of BRD4 and lysine acetylation level decreases in JQ1 exposed MII oocytes

**Fig. 5:**
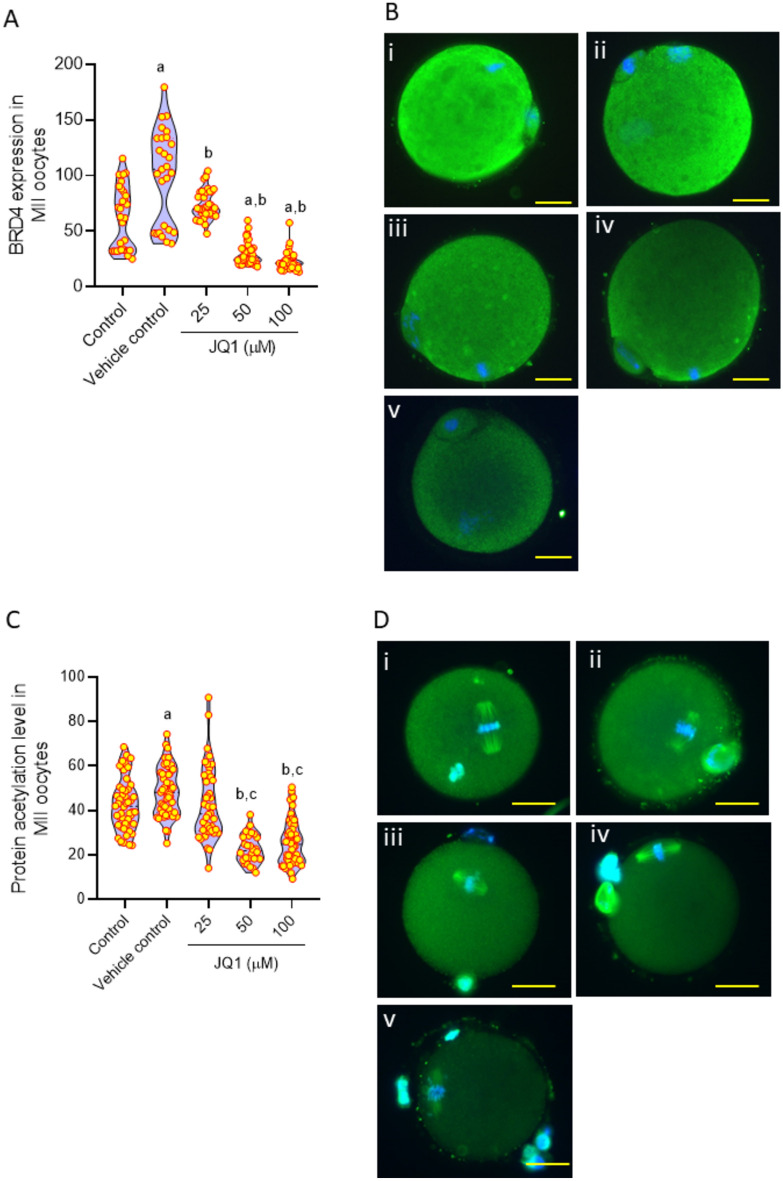
(**A**) Effect of JQ1 on BRD4 expression in MII oocytes. ^a^p < 0.0001 compared to control; ^b^p < 0.0001 compared to vehicle control. (**B**) Representative images of oocytes showing BRD4 expression in MII oocytes assessed by immunofluorescence technique; (i) control, (ii) vehicle control, (iii) JQ1 25 µM, (iv) JQ1 50 µM and, (v) JQ1 100 µM (magnification 400x). The scale bar represents 20 µm. Number of oocytes in Control = 30; Vehicle control = 27; JQ1 25 µM = 36; JQ1 50 µM = 47; JQ1 100 µM = 37. (**C**) Effect of JQ1 on lysine acetylation level in MII oocytes assessed by immunofluorescence technique. ^a^p < 0.05, ^b^p < 0.0001 compared to control; ^c^p < 0.0001 compared to vehicle control. (**D**) Representative images of M II oocytes with lysine acetylation expression level in (i) control, (ii) vehicle control, (iii) JQ1 25 µM, (iv) JQ1 50 µM, and (v) JQ1 100 µM (magnification 400x). The scale bar represents 20 µm. Number of oocytes in Control = 48; Vehicle control = 51; JQ1 25 µM = 43; JQ1 50 µM = 32; JQ1 100 µM = 46.

No significant change in BRD4 expression was observed, in MII oocytes exposed to the lowest concentration of JQ1 (25 µM) compared to control. However, at 50 and 100 µM concentrations of JQ1, the expression significantly decreased (p < 0.0001) in MII oocytes (Fig. 5A,B). Protein lysine acetylation level did not differ between the MII oocytes of control and vehicle control groups. Lysine acetylation levels remained unchanged at 25 µM concentration of JQ1, but significantly decreased in MII oocytes exposed to 50 and 100 µM of JQ1 compared to the oocytes of control and vehicle control groups (p < 0.0001, Fig. 5C,D).

### The detrimental effects of JQ1 on oocytes are mediated through oxidative stress

**Fig. 6:**
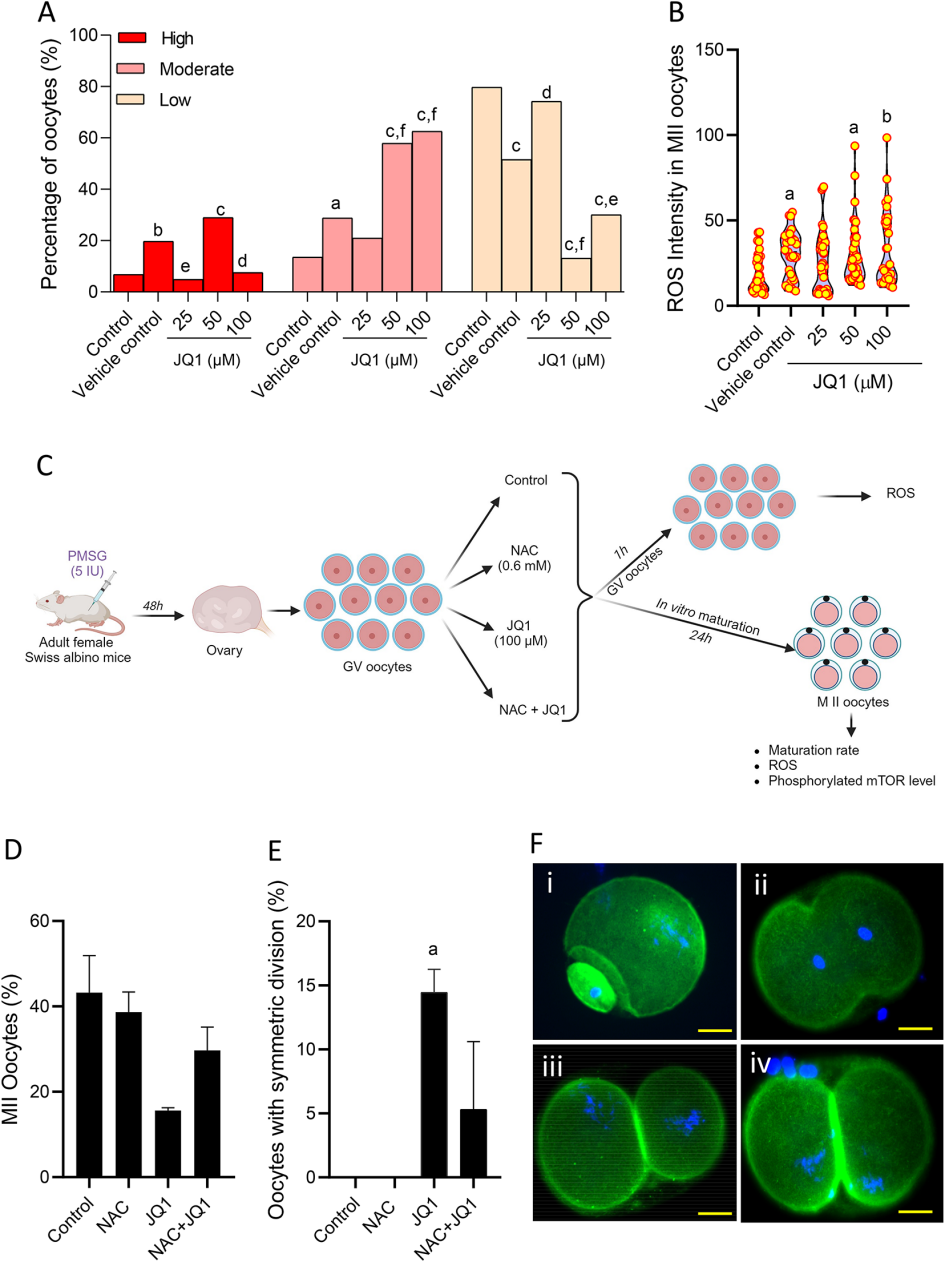
Effect of JQ1 exposure on the intracellular ROS assessed by staining with DCFH-DA in MII oocytes at 24 h after IVM. (**A**) Graph depicting the percentage of oocytes with high, moderate, and low intracellular ROS levels. ^a^p < 0.05, ^b^p < 0.01, ^c^p < 0.0001 compared to control; ^d^p < 0.05, ^e^p < 0.01, ^f^p < 0.0001 compared to vehicle control. (**B**) Mean intensity of intracellular ROS level in MII oocytes exposed to various concentrations of JQ1 in vitro. ^a^p < 0.05, ^b^p < 0.01 compared to control and NAC. Number of oocytes in Control = 34; Vehicle control = 43; JQ1 25 µM = 36; JQ1 50 µM = 36; JQ1 100 µM = 33. (**C**) Schematic representation to show the effect of N-Acetyl cysteine (NAC, 0.6 mM) on JQ1-induced cytoplasmic defects in mouse oocytes under in vitro conditions (Created with BioRender.com). (**D**) Effect of JQ1 on the maturation (M II) rate in JQ1 exposed oocytes cultured in the presence of NAC. Number of oocytes in Control = 107; NAC = 109; JQ1 = 167; NAC + JQ1 = 144. (**E**) Effect of NAC on the JQ1-induced symmetric cytokinesis in MII stage oocytes. ^a^p < 0.05 compared to control. (**F**) Representative images of MII oocytes exhibiting (i) asymmetric, and (ii-iv) symmetric cytokinesis. Oocytes were stained with phalloidin and counterstained with DAPI (magnification 400x). The scale bar represents 20 µm.

MII oocytes from JQ1-exposed groups exhibited elevated ROS levels compared to oocytes from the control groups. The percentage of MII oocytes with high and moderate ROS levels was significantly higher (p < 0.0001) in 50 µM concentrations compared to the control group (Fig. 6A). Similarly, at 100 µM concentration of JQ1, the percentage of MII oocytes with moderate ROS level was significantly higher (p < 0.0001) compared to the control group. In parallel, the percentage of oocytes with low intracellular ROS levels was significantly lower (p < 0.0001) in both 50 and 100 µM concentrations compared to the control group. Further, when we quantified the intracellular ROS level in MII oocytes (Fig. 6B), significantly higher intensity was observed in MII oocytes derived from GV oocytes exposed to 50 and 100 µM concentrations of JQ1 (p < 0.05 compared to control).

**Fig. 7:**
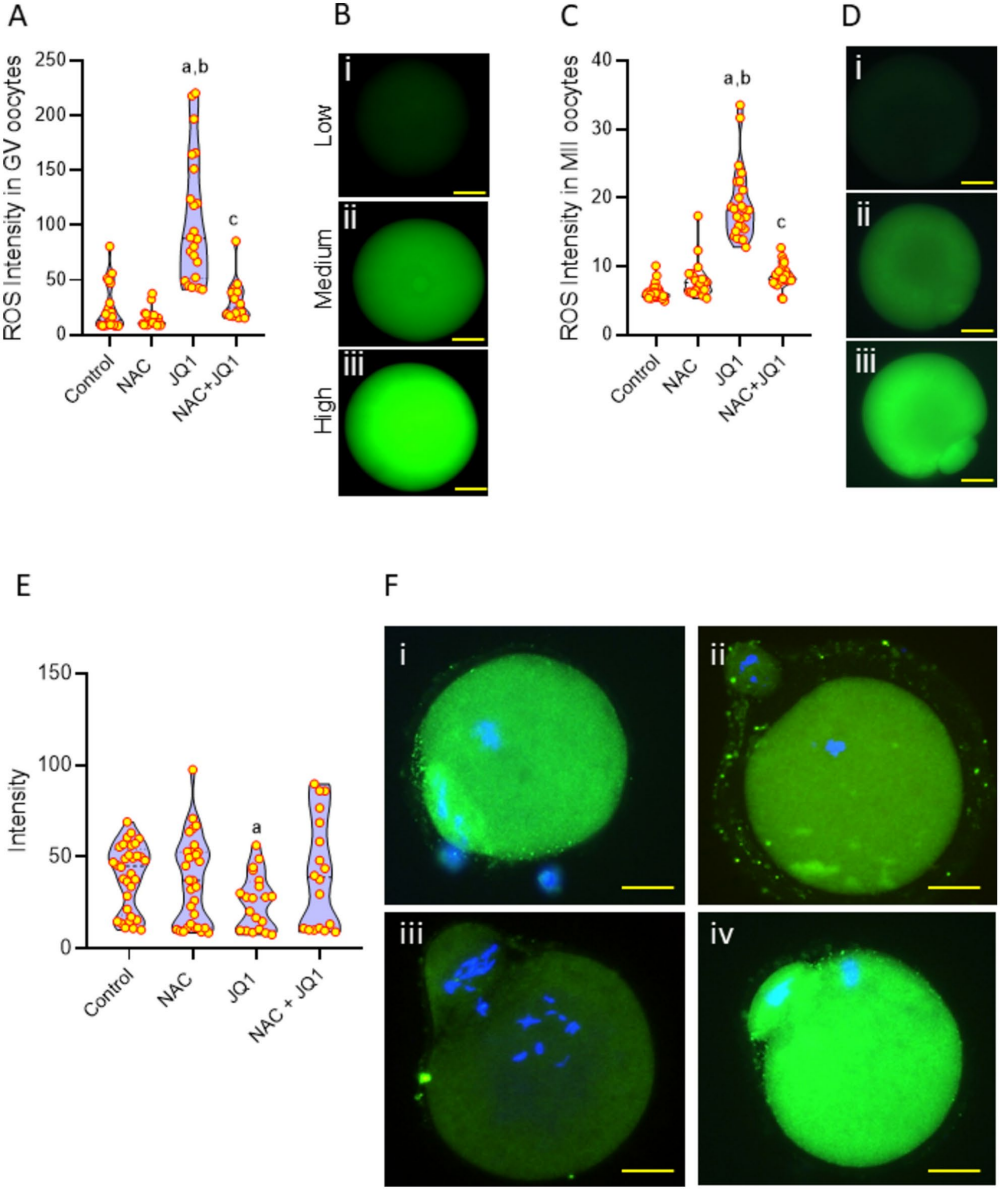
(**A**) Effect of NAC on the JQ1-induced ROS in GV oocytes. ^a^p < 0.0001 compared to control; ^b^p < 0.0001 compared to NAC; ^c^p < 0.0001 compared to JQ1. (**B**) Representative images of GV stage oocytes with low, medium and high intracellular ROS level (magnification 400x). The scale bar represents 20 µm. Number of oocytes in Control = 23; NAC = 21; JQ1 = 14; and NAC + JQ1 = 27. (**C**) Effect of NAC on the JQ1-induced ROS in MII stage oocytes. ^a^p < 0.0001 compared to control; ^b^p < 0.0001 compared to NAC; ^c^p < 0.0001 compared to JQ1. (**D**) Representative images of MII stage oocytes with low, medium and high intracellular ROS level (magnification 400x). The scale bar represents 20 µm. Number of oocytes in Control = 66; NAC = 69; JQ1 = 50; NAC + JQ1 = 70. (**E**) Effect of NAC on phosphorylated mTOR (p-mTOR) expression level assessed by immunofluorescence technique in MII oocytes exposed to JQ1. ^a^p < 0.05 compared to control. (**F**) Representative images of p-mTOR expression in MII oocytes of (i) control, (ii) NAC, (iii) JQ1, (iv) NAC + JQ1 (magnification 400x). The scale bar represents 20 µm. Number of oocytes in Control = 34; NAC = 31; JQ1 = 33; NAC + JQ1 = 25.

To investigate if the JQ1-induced decrease in oocyte maturation rate in vitro is mediated through oxidative stress, we cultured the oocytes in IVM medium with or without antioxidant NAC (Fig. 6C). The JQ1-exposed oocytes had lower maturation potential (Fig. 6D) and a higher percentage of MII oocytes with symmetric division compared to the control and NAC groups (p < 0.05, Fig. 6E,F). The presence of NAC in the culture medium rescued the oocytes from JQ1-induced adverse effects as evident from non-significant increase in the maturation rate and decrease in the percentage of MII oocytes with symmetric division. Further, when we assessed the effect of short-term exposure (1 h) of JQ1 on GV oocytes, a significant increase in ROS level was observed in GV oocytes exposed to 100 µM of JQ1 compared to the control and NAC groups (p < 0.0001). NAC resulted in a significant decrease in ROS level compared to the JQ1 group (p < 0.0001) (Fig. 7A,B).

Similarly, when we assessed the consequences of long-term exposure (24 h) on oocytes, the intensity of ROS in JQ1-exposed MII oocytes was significantly higher compared to the control and NAC groups (p < 0.0001). However, significant decreases in ROS levels were observed in oocytes cultured with JQ1 in combination with NAC compared to JQ1 alone-exposed oocytes (p < 0.0001). The presence of NAC in combination with JQ1 significantly reduced ROS levels in MII oocytes compared to JQ1 alone-exposed oocytes (Fig. 7C,D).

### The presence of JQ1 decreased phosphorylated mTOR (p-mTOR) levels in MII oocytes

Expression of phosphorylated mTOR was comparable in oocytes of control and NAC groups. A significant decrease (p < 0.05 compared to control) in the p-mTOR level was observed in JQ1 exposed oocytes compared to control and NAC groups (Fig. 7E,F). The presence of NAC in the JQ1 group resulted in a non-significant increase in p-mTOR level.

### JQ1 administration inhibited ovulation and altered OCC quality in mice

**Fig. 8:**
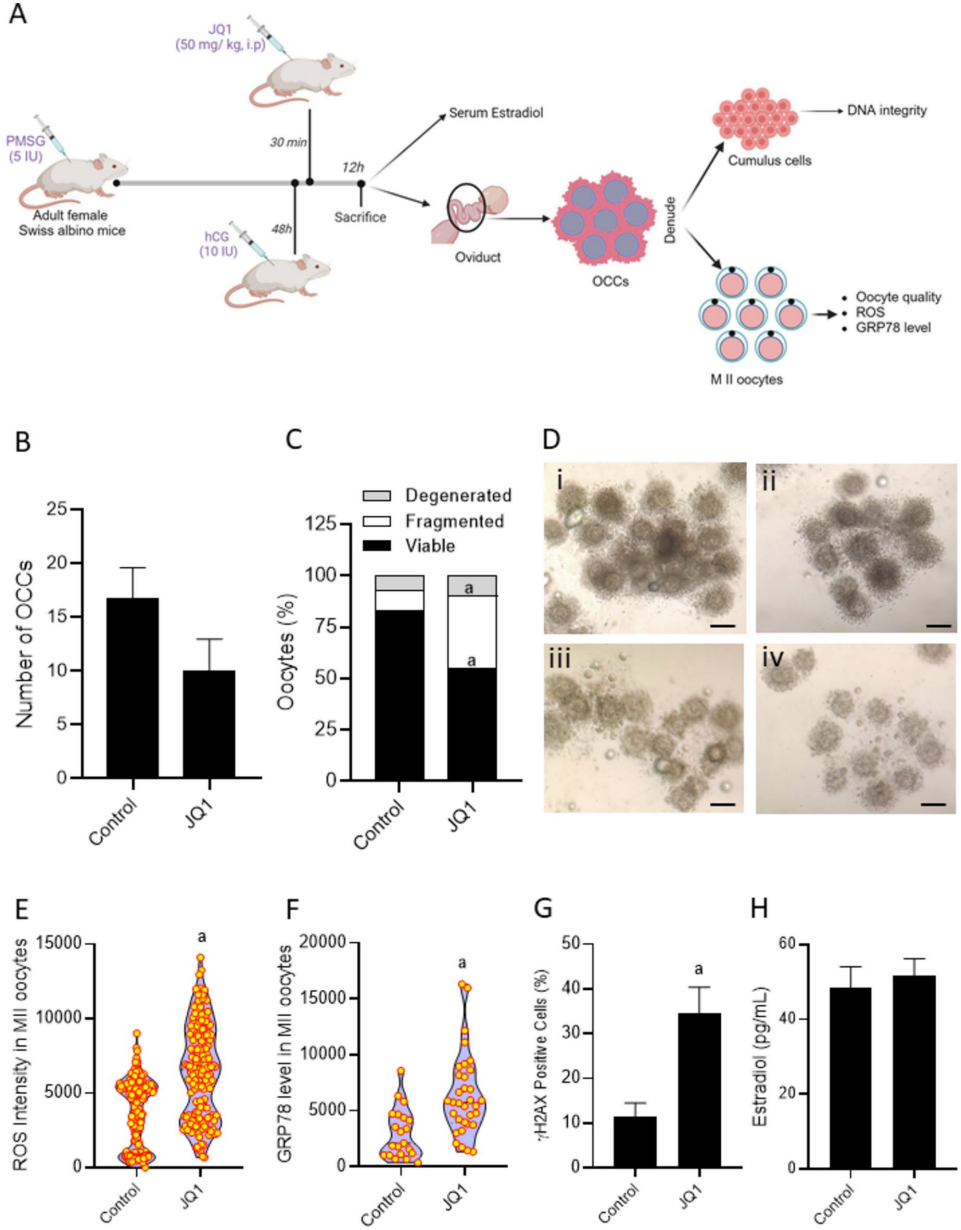
(**A**) Schematic representation (Created with BioRender.com) to show the experimental outline to understand the effect of JQ1 administration (50 mg/kg, i.p.) on ovulation in Swiss albino mice primed with PMSG (5 IU) and hCG (10 IU). (**B**) Number of oocyte cumulus complexes (OCCs) retrieved from control and JQ1 treated mice (n = 6). (**C**) Quality of MII oocytes retrieved from control and JQ1 treated mice. ^a^p < 0.0001 compared to control. (**D**) Representative images of OCCs collected from the oviduct of (i & ii) control and (iii & iv) JQ1 treated mice. The scale bar represents 200 µm. (**E**) Intracellular ROS level in MII oocytes retrieved from control and JQ1 administered mice assessed by DCFHDA staining method. ^a^p < 0.0001 compared to control. Number of oocytes in Control = 181, JQ1 = 154. (**F**) GRP78 expression assessed by immunofluorescence technique in M II oocytes retrieved from control and JQ1 administered mice. ^a^p < 0.001 compared to control. Number of oocytes in Control = 21, JQ1 = 33*.* (**G**) γH2AX foci in cumulus cells collected from control and JQ1 administered mice assessed by immunofluorescence technique. ^a^p < 0.001 compared to control. Effect of JQ1 on (**H**) Serum estradiol level in mice primed with PMSG and hCG at 12 h after hCG administration (n = 6*).*

Administering JQ1 at 30 min after hCG injection resulted in a non-significant decrease in the number of ovulated oocytes compared to the control group (Fig. 8A,B). Further, we observed a significant decrease in the percentage of viable oocytes (p < 0.0001) and a significant increase in the percentage of (p < 0.0001) fragmented oocytes in JQ1-administered mice compared to the control (Fig. 8C). The oocyte-cumulus complexes (OCCs) from the JQ1 group displayed very scanty and dispersed cumulus cells surrounding the oocytes (Fig. 8D). Furthermore, the oocytes exhibited a significant increase in intracellular ROS levels (p < 0.0001, Fig. 8E) and GRP78 expression (p < 0.001, Fig. 8F) compared to oocytes from control mice. The cumulus cells were characterized by the presence of high percentage (p < 0.001) of γH2AX-positive cells in the JQ1 group compared to the control (Fig. 8G). However, the serum estradiol (Fig. 8H) levels in mice administered with JQ1 was unaltered.

### Computational studies

Genes targeted by JQ1 with high binding scores were selected for network biology analyses. For a systematic assessment of the biological functions within the target network, protein–protein interaction (PPI) networks were formulated using the Cytoscape software, utilizing information sourced from the STRING database. The resulting PPI network comprised 112 nodes and 1600 edges, with nodes representing genes and edges denoting gene associations (Fig S1, Supplementary information).

To probe into the potential functions of the target gene network, Gene Ontology (GO) and pathway enrichment analyses were conducted employing ClueGO. The foremost significantly enriched GO term in the Biological Process category was “mitotic cell cycle,” encompassing 30.72% of total GO terms, followed by “cellular response to oxygen levels” (10.58%) and “cell cycle phase transition” (9.56%). Notable GO terms pertained to cell cycle-related regulatory pathways, cellular response to oxidative stress, and positive regulation of cell death (Fig S2, Supplementary information). Predominant molecular functions (Fig S3, Supplementary information) and cellular components (Fig S4, Supplementary information) associated with the target gene network included “chromosomal region” (33.33%) and regulation of transferase activity (45.95%).

In the KEGG pathway enrichment analysis, the primary pathways associated with the target gene network were identified. The top three enriched pathways were “prostate cancer” (60%), FoxO signalling pathway (7.2%), Cellular senescence (6.4%), and cellular components (Fig S5, Supplementary information). The enrichment outcomes suggest that the effects of JQ1 may be linked to the modulation of the aforementioned biological processes, primarily influencing cell cycle-related processes. Detailed results of the gene ontology and pathway enrichment analysis, along with the involved genes, are available in the supplementary file.

## Discussion

This is the first report to provide evidence that small molecule JQ1 disrupts the cytoplasmic organization and exerts an inhibitory effect on the nuclear maturation of oocytes. The altered distribution of mitochondria and cortical granules, impaired organization of actin and tubulin filaments, misaligned chromosomes, and symmetric cytokinesis during the second meiotic division collectively suggest that JQ1 significantly reduces the functional competence of the oocytes.

Earlier studies have used JQ1 as a potent non-hormonal chemotherapeutic agent^26^, specifically for endocrine-sensitive cancer types^27^. JQ1 has shown an inhibitory effect on the proliferation of murine embryonic^15^ and mesenchymal stem cells^28^. Further, administration of JQ1 to male mice has shown to inhibit the spermatogenesis by depleting the germ cell population in the adult mouse^13,14^. In line with earlier reports, in our study we observed maturation arrest in oocytes exposed to JQ1 during in vitro maturation, suggesting that JQ1 has anti-proliferative effects on female germ cells. Functional analysis of the JQ1-targeted gene network primarily implicated cell cycle pathways and cell phase transitions, indicating a significant role in disrupting cell cycle processes (Fig S2). Cell cycle inhibitory effects of JQ1 has been demonstrated by earlier studies^29^, which was strongly associated with inhibition of lysine acetylation process^30,31^. A similar association of oocyte maturation arrest with decreased lysine acetylation has been observed in our study.

The unique asymmetric cytokinesis during the progression from MI to MII (extrusion of first polar body) is the characteristic feature of the mammalian oocytes. Exposure of oocytes to JQ1 disrupted the asymmetric cytokinesis, which resulted in oocytes with polar body having atypical, large cytoplasmic volume, with 2-cell stage embryo-like appearance. Oocytes exposed to histone deacetylase inhibitors induce similar features in oocytes. Further, they were shown to trigger spindle deformities and chromosome misalignment in murine oocytes^30,32–34^. Symmetric cytokinesis and spindle migration are controlled by microtubules and microfilaments^35^. Polymerization of actin and tubulin filaments are regulated by post-translational modifications like lysine acetylation^36^. Our results are in agreement with earlier findings^30,32–34^, JQ1 exposed oocytes had lower protein lysine acetylation level, and defective actin and tubulin organization, collectively contributing to symmetric cytokinesis. Further, we observed that JQ1 exposure resulted in downregulation of phosphorylated mammalian target of rapamycin (p-mTOR), which is known to be associated with regulation of oocyte polarity, cortical granule distribution and spindle migration^37^. The molecular docking results indicated a strong affinity of JQ1 for mTOR (supplementary information) suggesting its possible involvement in mTOR pathway. Inactivation of mTOR pathway by JQ1 has been documented in ovarian cancer cells^38^ and hematologic cancers like multiple myeloma and primary effusion lymphoma^39^.

In metaphase II oocytes, highly condensed chromosomes are arranged in equatorial plates, the segregation of which is controlled by the meiotic spindle^40^. The histone acetylation process plays a critical role in chromosome segregation during oocyte maturation^34^. It is well documented that a decrease in acetylation affects chromosome condensation and segregation, ultimately resulting in aneuploidy as well as abnormal cell division^41^. The JQ1-exposed MII oocytes had a high incidence of spindle defects and chromosome misalignment and were associated with lower lysine acetylation levels. The molecular function and cellular component analyses conducted in this study clearly highlight the involvement of JQ1-targeted genes in spindle formation and microtubule binding, reinforcing the findings (Figs. S3 and S4). In addition, the computational data indicated that JQ1 has high affinity for the proteins involved in spindle organization such as LIMK1^42^, CDK1^43^, HDAC6^30,32,33^, ROCK1^44^, SIRT2^45^, ARF1^46^, and PLK1^43^. The high binding affinity suggests a potential cause for the disrupted actin distribution, spindle organization and increased chromosome misalignment as well as increased percentage of symmetric division in JQ1 exposed oocytes.

Endoplasmic reticulum (ER) is an important cytoplasmic organelle which help in the maintenance of calcium homeostasis and synthesis of membrane protein and lipids. The ER dysfunction is shown to be associated with female infertility^47^. GRP78 is a master regulator of ER stress, due to its ability to control the activation of unfolded protein response signaling^48^. Oxidative stress has shown to cause ER stress thereby affecting the reproductive function^49^. ER stress homeostasis play a critical role in maintaining the oocyte quality^47^. Poor quality of MII oocytes retrieved from JQ1-administered animals could be due to increase in GRP78 expression as a result of elevated ROS in ovulated oocytes. IVM-derived MII oocytes are known to have elevated oxidative stress^50,51^, which is shown to affect nuclear maturation, meiotic resumption, altered mitochondrial function, poor fertilization potential^51^ and poor developmental potential^52^. ROS beyond physiological level has shown to affect the oocyte quality. Addition of antioxidants has proven to mitigate the detrimental effects of ROS and improve the oocyte quality and maturation^20,50^.

ER stress and oxidative stress are closely associated events, the elevated level of which reflects the poor oocyte quality (Sasaki et al., 2019). The second most enriched biological processes associated with JQ1-targeted genes involve responses to oxygen levels and oxidative stress, which aligns with our study’s findings (Fig S2). In our study, exposure of GV oocytes to JQ1, even for a short duration (1 h) under in vitro condition, was able to induce significant oxidative stress. Elevated ROS level induced by JQ1 in oocytes might have contributed to the poor maturation potential and other cytoplasmic defects, as supplementation of NAC, a precursor of reduced glutathione (GSH) and, a potent antioxidant to IVM medium not only decreased the ROS level (both in short term and long term exposure groups), but also improved the nuclear maturation. Beneficial effect of NAC with concentration ranging between 0.6 mM and 1.5 mM as an antioxidant, has shown to improve oocyte maturation and quality as well as embryo quality by reducing ROS level, improving mitochondrial function, cell viability and, decreasing DNA damage and cell apoptosis^20^. ROS–mediated changes in lysine acetylation level and mTOR signaling has been demonstrated by earlier studies^53,54^. The elevated p-mTOR level, decreased percentage of oocytes with symmetric cytokinesis, and increased percentage of nuclear maturation in oocytes cultured in the presence of NAC, indicates that the detrimental effects of JQ1 on the nuclear maturation and cytoplasmic organization of oocytes is mediated through oxidative stress.

Further, to understand if the JQ1 had anovulatory effect in females, we administered JQ1 to female mice post gonadotropin administration. JQ1 did not alter the ovulation in the animals, however, as observed in in vitro experiments, we observed a significant increase in the percentage of poor-quality oocytes. JQ1 administration did not have any impact on serum estradiol level and ovulation. A previous report by Matzuk et al.^13^, have shown that intraperitoneal administration of 50 and 75 mg/kg JQ1 to male mice for 2 months did not result in any changes in the reproductive hormones. Our findings agree with this report.

Cumulus cells play a pivotal role providing physical and metabolic support and, create suitable microenvironment during oocyte development and maturation^55^. Increased apoptosis in cumulus cells has been associated with poor developmental potential of oocytes, impaired fertilization and pregnancy outcomes^56^. Interdependence of the oocyte and cumulus cells suggest that proper functioning of cumulus cells is essential to ensure the survival and subsequent maturation of the oocytes^55^. A significant increase in the DNA damage in the cumulus cells was observed in the JQ1 administered mice compared to control mice, indicating the compromised quality of the ovulated oocytes. Consistent with our experimental results, the functional analysis of the JQ1 target network also indicated the involvement of target genes in apoptosis and cellular senescence (Figs. S2 and S5). The data obtained from in vivo experiment suggest that JQ1 administration affects the oocyte quality in mice which could ultimately compromise the fertilizing potential as well as embryo development potential of the oocytes.

In conclusion, for the first time we demonstrated the effect of in vivo and in vitro exposure of oocytes to JQ1. The application of a network biology approach and the Gene Ontology (GO) enrichment analysis provided additional validation, thereby strengthening the observed effects of JQ1 exposure on oocytes, both in vivo and in vitro. We confirm that JQ1 elevated intracellular ROS levels in oocyte which compromised the oocyte quality. JQ1 does not interfere with the endocrine levels indicating the non-hormonal activity of the drug. Disrupted organelle and cytoskeleton distribution and misaligned chromosome can increase the aneuploidy in the JQ1 exposed oocytes, in the present study we did not perform any aneuploidy assessment to confirm. Interference of the drug with cortical reorganisation and cumulus distribution in oocytes, can result in compromised fertilizing ability, however, further studies are required to validate the same. Based on the data obtained, JQ1 does seem to have a potential as a non-hormonal female contraceptive, however, further studies are needed to confirm its activity on fertilization and embryo outcomes.

## Supplementary Information

Supplementary Information 1.

Supplementary Information 2.

## Data Availability

All the data generated from this study are included in the manuscript and in the supplementary information.
