# Supplementary material for: Bromodomain and extraterminal protein inhibitor JQ1 induces maturation arrest and disrupts the cytoplasmic organization in mouse oocytes under in vitro conditions
Source: Sci Rep. 2025 Apr 18;15:13448. doi: 10.1038/s41598-025-96687-z (PMC12008386; doi:10.1038/s41598-025-96687-z)
Supplement: Supplementary file 1 — Supplementary Information 1. [file 41598_2025_96687_MOESM1_ESM.docx]

Bromodomain and Extraterminal Protein Inhibitor JQ1 Induces Maturation Arrest and Disrupts the Cytoplasmic Organization in Mouse Oocytes under *In Vitro* Conditions

Keerthana Sandesh Suvarna^1^, Jyolsna Ponnaratta Kunhiraman^2^, Vanishree Vasave Madhvacharya^2^, Sandhya Kumari^2^, Navami Krishna^3^, Suresh PS^3^, Rajanikant GK^3^, Srinivas Mutalik^4^, Nadeem G Khan^5^, Shama Prasad K^5^, Prashanth Modi^6^, Thottethodi Subrahmanya Keshava Prasad^6^, Satish Kumar Adiga^1^, Guruprasad Kalthur^2#^

^1^Centre for Excellence in Clinical Embryology, Department of Reproductive Science, Kasturba Medical College, Manipal, Manipal Academy of Higher Education, Manipal 576104, India

^2^Division of Reproductive Biology, Department of Reproductive Science, Kasturba Medical College, Manipal, Manipal Academy of Higher Education, Manipal 576104, India

^3^School of Biotechnology, National Institute of Technology, Calicut 673601, Kerala, India

^4^Department of Pharmaceutics, Manipal College of Pharmaceutical Sciences, Manipal Academy of Higher Education, Manipal 576104, India

^5^Manipal School of Life Sciences, Manipal Academy of Higher Education, Manipal 576104, India

^6^Center for Systems Biology and Molecular Medicine, Yenepoya Research Center, Yenepoya (Deemed to be University), Mangalore -575018, India

^#^Author for correspondence

Dr Guruprasad Kalthur,

Professor and Head,

Department of Reproductive Science,

Head, Division of Reproductive Biology

Kasturba Medical College, Manipal,

Manipal Academy of Higher Education, Manipal 576104, India

Email: [guru.kalthur@manipal.edu](mailto:guru.kalthur@manipal.edu)

**Methodology for computational studies:**

Protein–ligand docking is a crucial aspect of computer-aided drug discovery (CADD), widely utilized for predicting binding modes and affinities. Blind docking, performed on the entire protein surface, is valuable for identifying potential interactions in unknown binding regions. We have used CB-Dock, a blind docking tool that enhances accuracy by predicting binding regions through a curvature-based cavity detection approach (Liu et al., 2020). CB-Dock employs Autodock Vina for docking and achieves a success rate of approximately 70% for top-ranking poses with root mean squared deviation within 2 Å from the X-ray crystal structure position.

The Protein Data Bank (PDB) was used to download 3D X-ray diffraction structures of proteins important to the mechanism of symmetric division. Following that, the structural configurations of both the target protein and the JQ1 query ligand were used in blind docking simulations, with a parameter value of five used to determine the expected binding sites. The goal of these simulations was to predict JQ1 binding affinities with different proteins. These predictions were based on the computational procedure's docking scores and were expressed as -kcal/mol.

**Table S1: Blind docking of JQ1 with human LIMK1 (PDB ID: 3S95; Uniprot ID: P53667). The table listed Vina scores, cavity sizes, docking centers, and sizes of the top 5 predicted cavities.**

| **Vina score** | **Cavity size** | **Center** | | | **Size** | | |
| --- | --- | --- | --- | --- | --- | --- | --- |
|  |  | **x** | **y** | **z** | **x** | **y** | **z** |
| -9.9 | 231 | 24 | 29 | 65 | 22 | 22 | 22 |
| -7.6 | 157 | 24 | 8 | 31 | 22 | 22 | 22 |
| -7.5 | 563 | 25 | 38 | 51 | 22 | 22 | 22 |
| -7.5 | 465 | 15 | 7 | 34 | 22 | 22 | 22 |
| -7.5 | 296 | 16 | 21 | 66 | 22 | 22 | 22 |

**Table S2: Blind docking of JQ1 with LIMK2 (PDB ID: 7QHG; Uniprot ID: P53671). The table listed Vina scores, cavity sizes, docking centers, and sizes of the top 5 predicted cavities.**

| **Vina score** | **Cavity size** | **Center** | | | **Size** | | |
| --- | --- | --- | --- | --- | --- | --- | --- |
|  |  | **x** | **y** | **z** | **x** | **y** | **z** |
| -7.8 | 7399 | 16 | -12 | 96 | 35 | 34 | 33 |
| -6.8 | 171 | 27 | -22 | 129 | 22 | 22 | 22 |
| -6.2 | 5218 | 46 | -13 | 109 | 30 | 35 | 22 |
| -6 | 1016 | 39 | -23 | 117 | 22 | 22 | 22 |
| -6 | 228 | 45 | 8 | 86 | 22 | 22 | 22 |

**Table S3: Blind docking of JQ1 with ARF1*GDP*Sec7 complex (PDB ID: 1RE0; Uniprot ID: P84077). The table listed Vina scores, cavity sizes, docking centers, and sizes of the top 5 predicted cavities.**

| **Vina score** | **Cavity size** | **Center** | | | **Size** | | |
| --- | --- | --- | --- | --- | --- | --- | --- |
|  |  | **x** | **y** | **z** | **x** | **y** | **z** |
| -8.2 | 272 | 30 | 0 | 49 | 22 | 22 | 22 |
| -7.4 | 1347 | 42 | 7 | 52 | 22 | 22 | 22 |
| -6.8 | 7833 | 39 | 9 | 38 | 32 | 35 | 22 |
| -6.5 | 279 | 34 | 32 | 54 | 22 | 22 | 22 |
| -4.7 | 188 | 32 | -14 | 33 | 22 | 22 | 22 |

**Table S4: Blind docking of JQ1 with CDK1/CKS2 complex [PDB ID: 6GU6; Uniprot ID: P06493 (CDK1) and K9J4F7 (CKS2)]. The table listed Vina scores, cavity sizes, docking centers, and sizes of the top 5 predicted cavities.**

| **Vina score** | **Cavity size** | **Center** | | | **Size** | | |
| --- | --- | --- | --- | --- | --- | --- | --- |
|  |  | **x** | **y** | **z** | **x** | **y** | **z** |
| -7.3 | 735 | 22 | 20 | -1 | 22 | 22 | 22 |
| -7 | 653 | 21 | 10 | 7 | 22 | 22 | 22 |
| -6.8 | 424 | 20 | 10 | 32 | 22 | 22 | 22 |
| -6.6 | 383 | 6 | 10 | 9 | 22 | 22 | 22 |
| -6.6 | 360 | 11 | 5 | -7 | 22 | 22 | 22 |

**Table S5: Blind docking of JQ1 with PLK-1 polo-box domain (PDB ID: 7MX1; Uniprot ID: P53350). The table listed Vina scores, cavity sizes, docking centers, and sizes of the top 5 predicted cavities.**

| **Vina score** | **Cavity size** | **Center** | | | **Size** | | |
| --- | --- | --- | --- | --- | --- | --- | --- |
|  |  | **x** | **y** | **z** | **x** | **y** | **z** |
| -7.5 | 232 | 110 | -6 | 76 | 22 | 22 | 22 |
| -7.2 | 922 | 128 | -2 | 73 | 22 | 22 | 22 |
| -6.9 | 990 | 119 | 5 | 56 | 22 | 22 | 22 |
| -6.7 | 881 | 111 | -5 | 18 | 22 | 22 | 22 |
| -6.7 | 753 | 120 | -7 | 41 | 22 | 22 | 22 |

**Table S6: Blind docking of JQ1 with HDAC6 zinc-finger ubiquitin binding domain (PDB ID: 8G44; Uniprot ID: Q9UBN7). The table listed Vina scores, cavity sizes, docking centers, and sizes of the top 5 predicted cavities.**

| **Vina score** | **Cavity size** | **Center** | | | **Size** | | |
| --- | --- | --- | --- | --- | --- | --- | --- |
|  |  | **x** | **y** | **z** | **x** | **y** | **z** |
| -6.9 | 296 | 2 | 2 | 11 | 22 | 22 | 22 |
| -6.1 | 25 | -3 | -8 | 11 | 22 | 22 | 22 |
| -5.6 | 50 | 17 | -6 | 12 | 22 | 22 | 22 |
| -5.5 | 157 | 11 | -1 | 19 | 22 | 22 | 22 |
| -5.5 | 62 | 9 | -15 | 6 | 22 | 22 | 22 |

**Table S7: Blind docking of JQ1 with human mTOR (PDB ID: 4JT5; Uniprot ID: P42345). The table listed Vina scores, cavity sizes, docking centers, and sizes of the top 5 predicted cavities.**

| **Vina score** | **Cavity size** | **Center** | | | **Size** | | |
| --- | --- | --- | --- | --- | --- | --- | --- |
|  |  | **x** | **y** | **z** | **x** | **y** | **z** |
| -9 | 4038 | -6 | 3 | -78 | 31 | 35 | 22 |
| -8.9 | 7316 | 63 | -36 | -25 | 32 | 35 | 22 |
| -8 | 1752 | 45 | -40 | -32 | 22 | 32 | 28 |
| -7.2 | 1319 | 80 | 6 | -47 | 22 | 22 | 22 |
| -7.2 | 1270 | 11 | -39 | -57 | 22 | 22 | 22 |

**Table S8: Blind docking of JQ1 with MAPK1 (PDB ID: 4ZZN; Uniprot ID: P28482). The table listed Vina scores, cavity sizes, docking centers, and sizes of the top 5 predicted cavities.**

| **Vina score** | **Cavity size** | **Center** | | | **Size** | | |
| --- | --- | --- | --- | --- | --- | --- | --- |
|  |  | **x** | **y** | **z** | **x** | **y** | **z** |
| -8.3 | 595 | -11 | 13 | 41 | 22 | 22 | 22 |
| -7.4 | 1678 | 9 | -1 | 54 | 28 | 22 | 22 |
| -6.5 | 297 | 0 | 15 | 36 | 22 | 22 | 22 |
| -6.3 | 209 | -14 | 1 | 38 | 22 | 22 | 22 |
| -4.8 | 231 | -14 | 10 | 67 | 22 | 22 | 22 |

**Table S9: Blind docking of JQ1 with human CFL1 (PDB ID: 5L6W; Uniprot ID: P23528). The table listed Vina scores, cavity sizes, docking centers, and sizes of the top 5 predicted cavities.**

| **Vina score** | **Cavity size** | **Center** | | | **Size** | | |
| --- | --- | --- | --- | --- | --- | --- | --- |
|  |  | **x** | **y** | **z** | **x** | **y** | **z** |
| -7.5 | 350 | -21 | 37 | 20 | 22 | 22 | 22 |
| -7.4 | 358 | -15 | 43 | 28 | 22 | 22 | 22 |
| -6.5 | 430 | -23 | 43 | 39 | 22 | 22 | 22 |
| -6.1 | 1104 | -16 | 5 | -4 | 22 | 22 | 22 |
| -4.9 | 2166 | -11 | 21 | 14 | 22 | 22 | 22 |

**Table S10: Blind docking of JQ1 with human SIRT2 (PDB ID: 5DY4; Uniprot ID: Q8IXJ6). The table listed Vina scores, cavity sizes, docking centers, and sizes of the top 5 predicted cavities.**

| **Vina score** | **Cavity size** | **Center** | | | **Size** | | |
| --- | --- | --- | --- | --- | --- | --- | --- |
|  |  | **x** | **y** | **z** | **x** | **y** | **z** |
| -9.1 | 4354 | -18 | -25 | 8 | 29 | 29 | 22 |
| -7.8 | 139 | -21 | -30 | 26 | 22 | 22 | 22 |
| -7.3 | 652 | 0 | -18 | -13 | 22 | 22 | 22 |
| -7.1 | 293 | -13 | -38 | 9 | 22 | 22 | 22 |
| -6.1 | 109 | -6 | -17 | -2 | 22 | 22 | 22 |

**Table S11: Blind docking of JQ1 with human ROCK1 (PDB ID: 3V8S; Uniprot ID: Q13464). The table listed Vina scores, cavity sizes, docking centers, and sizes of the top 5 predicted cavities.**

| **Vina score** | **Cavity size** | **Center** | | | **Size** | | |
| --- | --- | --- | --- | --- | --- | --- | --- |
|  |  | **x** | **y** | **z** | **x** | **y** | **z** |
| -7.5 | 1036 | -36 | -14 | 44 | 22 | 22 | 22 |
| -7.2 | 8684 | -47 | -33 | 19 | 33 | 35 | 35 |
| -6.8 | 2279 | -37 | -52 | -27 | 22 | 22 | 22 |
| -6.6 | 2054 | -55 | -45 | -21 | 22 | 28 | 22 |
| -6.6 | 1398 | -48 | -48 | 42 | 22 | 32 | 22 |

**Figure S1: Protein- Protein interaction network**

**
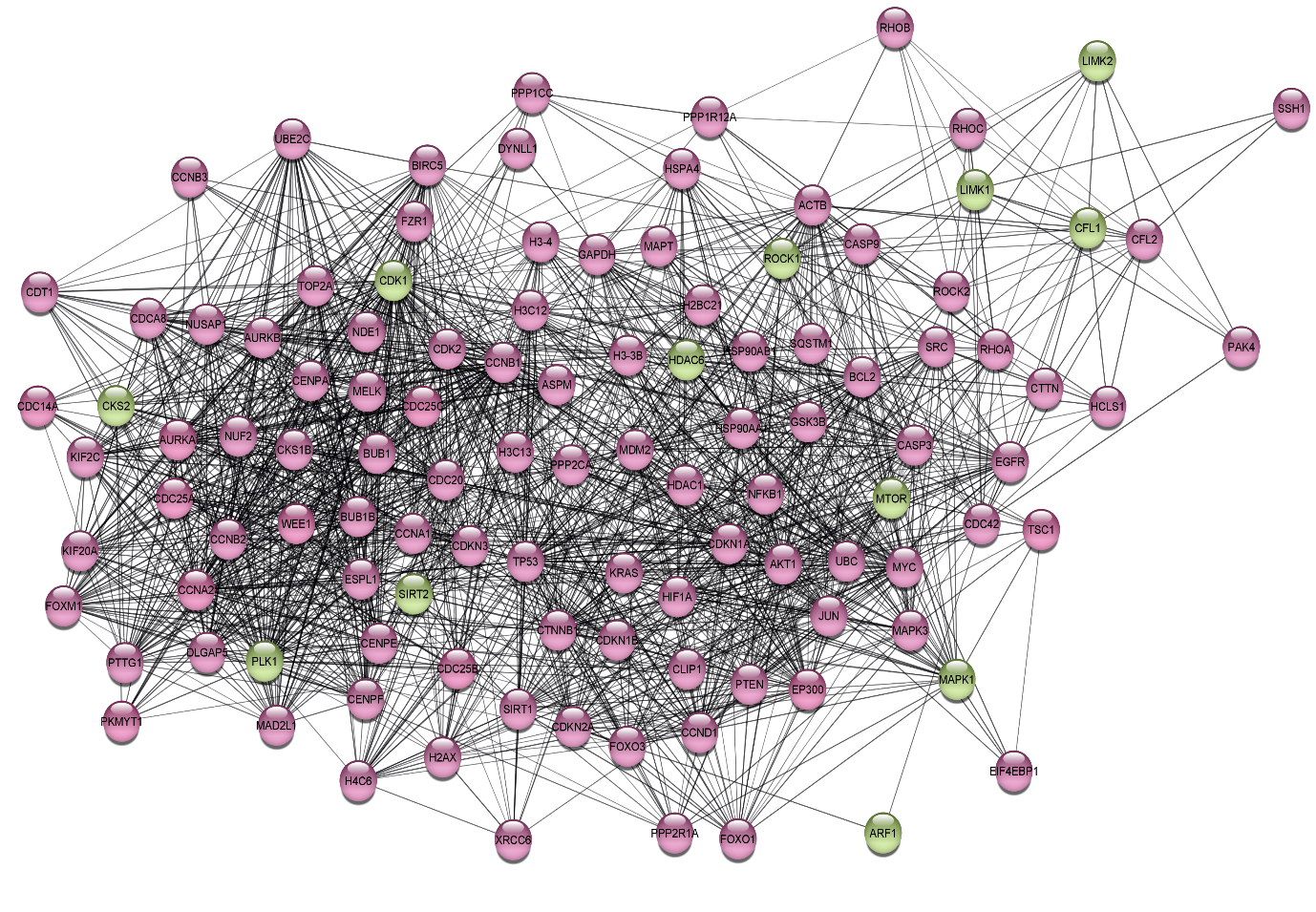
**

**Figure S2: Gene ontology (GO) biological process**


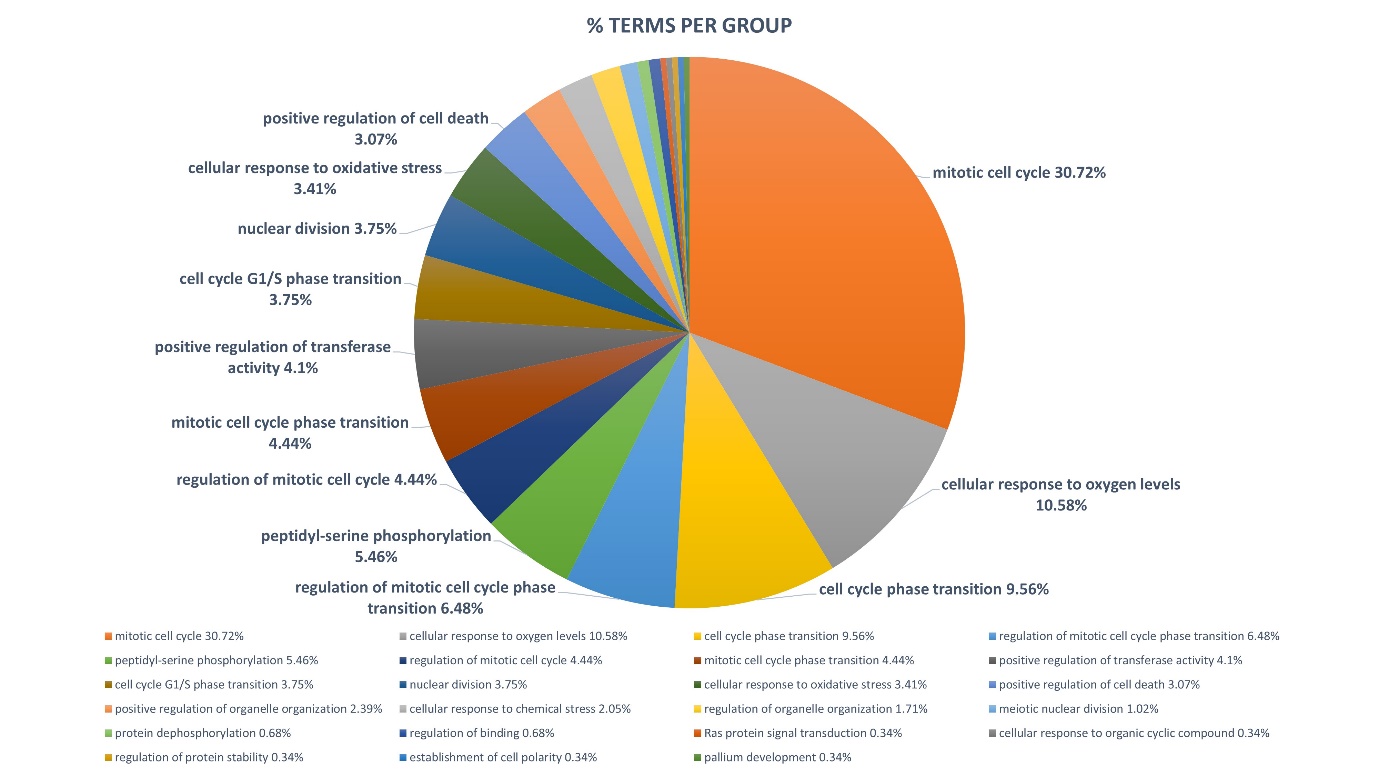


**Figure S3: Gene ontology molecular function**


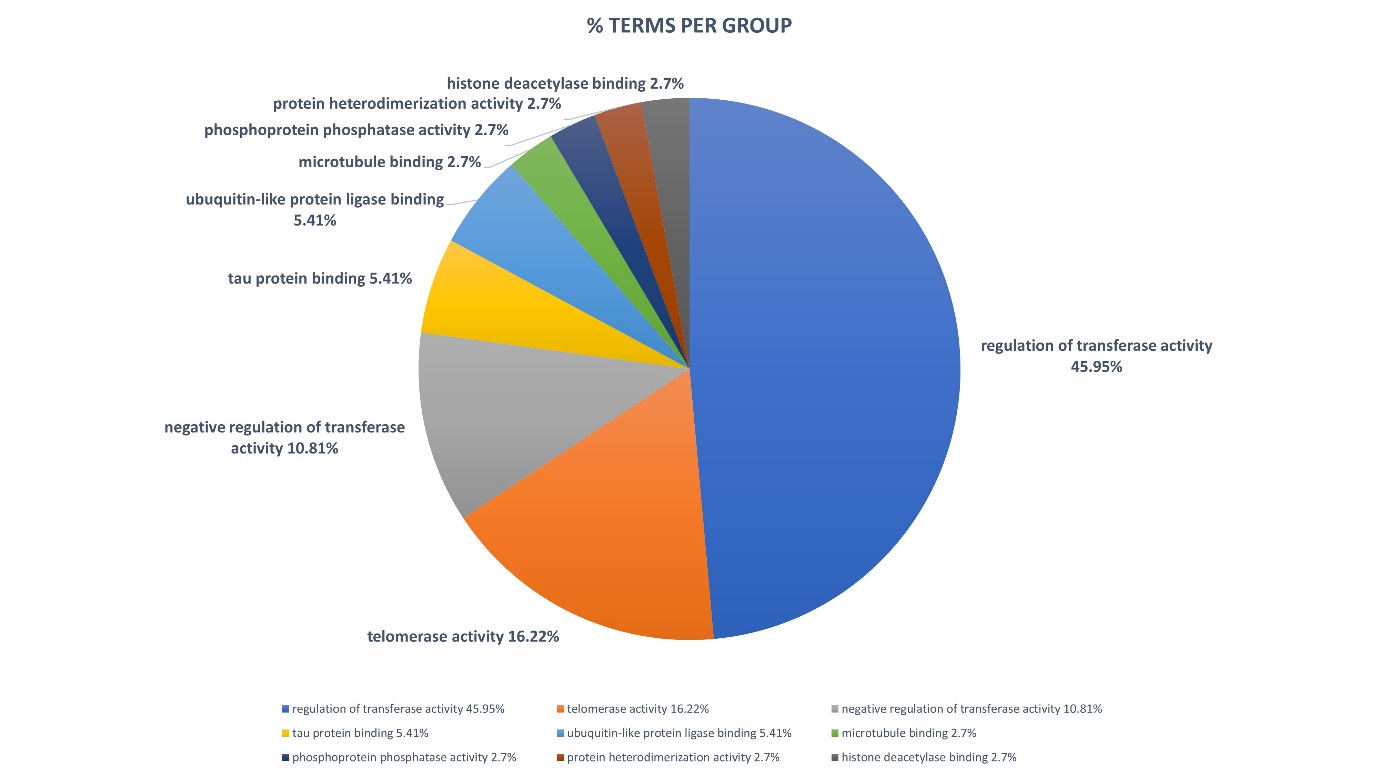


**Figure S4: Gene ontology cellular component**


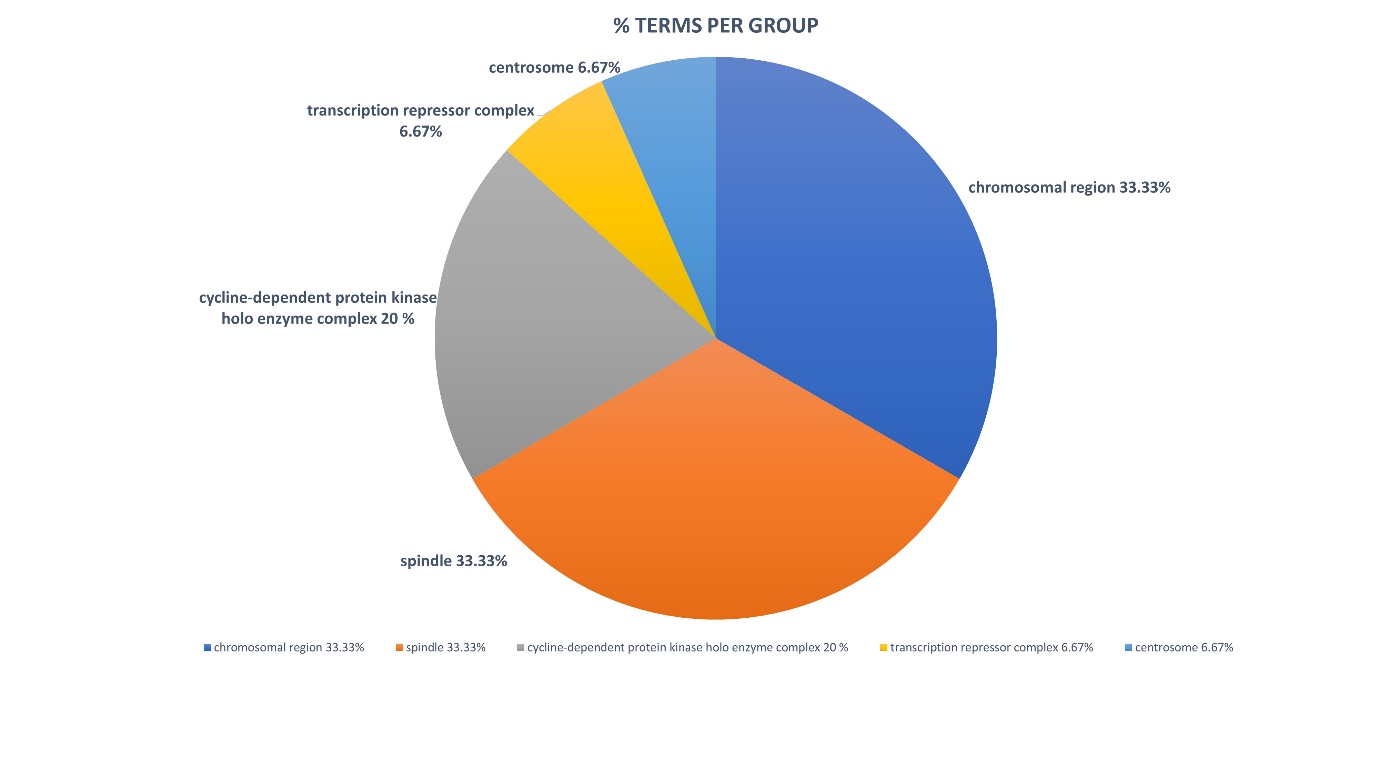


**Figure S5: KEGG pathway**


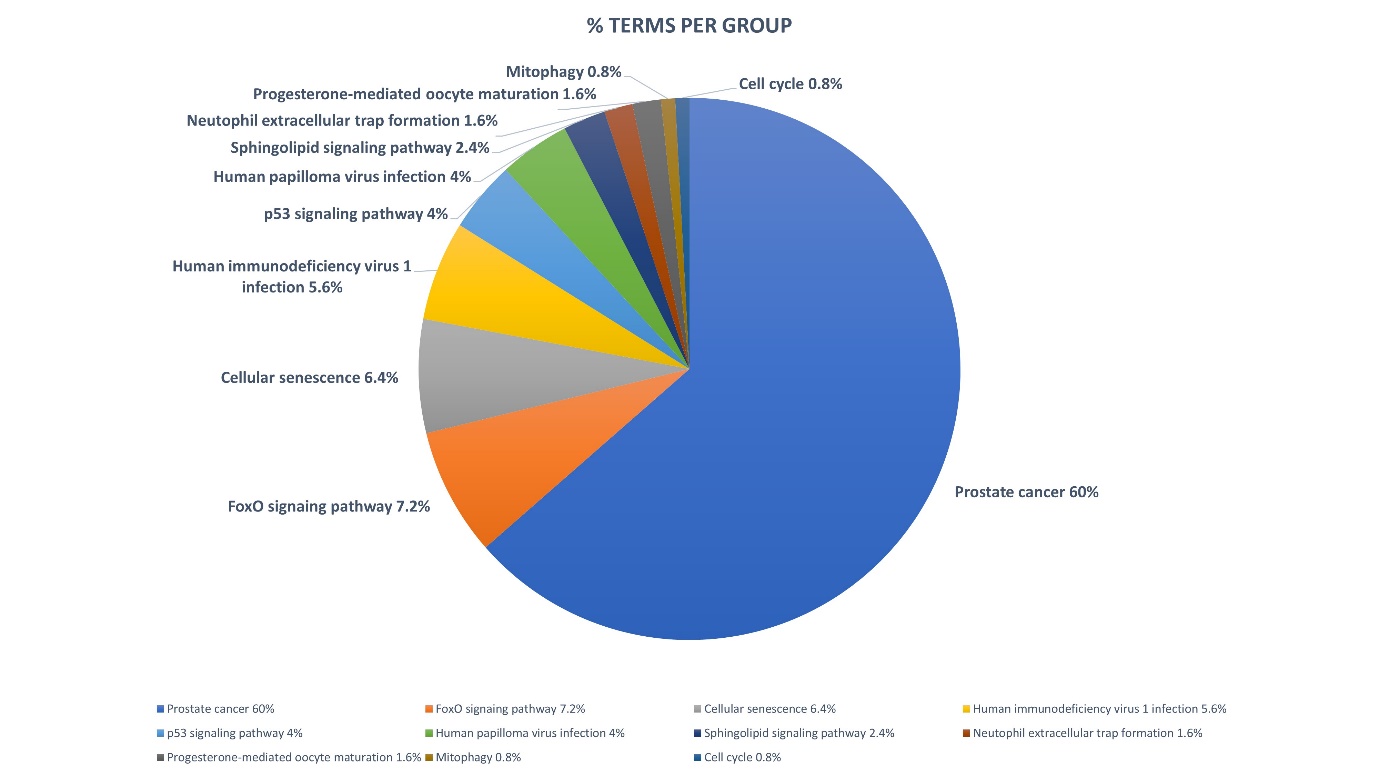


**Results:**

In the context of blind docking, we have chosen the top five cavities to conduct the docking process. Tables 1 through 11 present the docking scores and binding affinities of JQ1 with distinct proteins implicated in the process of symmetric division. Notably, the lowest docking score within each table is visually distinguished by a shaded grey highlight. It is important to note that a more negative Vina score is indicative of a heightened binding affinity, signifying the ligand's capacity to engage in spontaneous binding processes without necessitating energy expenditure.

**Discussion:**

In the context of blind docking studies, the discovery of JQ1 (a small molecule ligand) binding to multiple cavities within various distinct proteins implicated in the process of symmetric division presents a compelling and intriguing scenario. This observation warrants a detailed discussion in a scientific paper, highlighting the potential implications and future research directions.

The binding of JQ1 to multiple cavities across diverse proteins involved in symmetric division suggests a complex and multifaceted role for this ligand. Our findings suggest that JQ1 may possess a broader spectrum of activity by engaging with various binding sites across different proteins involved in symmetric division. This versatility could be advantageous in combating complex diseases where multiple protein targets are implicated. This finding may indicate that JQ1 could be a promising scaffold for the development of novel therapeutics targeting symmetric division, a process critical to the maintenance of tissue homeostasis and organ development.

The binding of JQ1 to multiple proteins may also imply that it could serve as a modulator of protein-protein interactions (PPIs) or allosteric regulator, which could have far-reaching consequences for the regulation of symmetric division. Further investigation into the specific binding sites and the effects of JQ1 on protein function and interactions could provide valuable insights into the molecular mechanisms governing symmetric division.

In conclusion, the discovery of JQ1 binding to multiple cavities within diverse proteins implicated in symmetric division presents a promising avenue for future research. The findings from blind docking studies warrant further experimental validation and functional investigation, which could lead to a better understanding of the molecular mechanisms governing symmetric division and potentially pave the way for the development of novel therapeutics targeting this process.

**Reference:**

Liu Y, Grimm M, Dai WT, Hou MC, Xiao ZX, Cao Y. CB-Dock: a web server for cavity detection-guided protein-ligand blind docking. Acta Pharmacol Sin. 2020 Jan;41(1):138-144. doi: 10.1038/s41401-019-0228-6. Epub 2019 Jul 1.
